# Generation and Enhancement of Persistent Nanoscale Magnetization in All‐Dielectric Metasurfaces by Optically Injected and Localized Free Carriers

**DOI:** 10.1002/advs.76635

**Published:** 2026-08-03

**Authors:** Shivaksh Rawat, Samyobrata Mukherjee, Gennady Shvets

**Affiliations:** ^1^ School of Applied and Engineering Physics Cornell University Ithaca New York USA

**Keywords:** Time‐varying photonics, Tunable mid‐IR metasurface

## Abstract

Time‐varying dielectric metasurfaces that support sharp optical resonances with nontrivial electromagnetic field distributions constitute a unique platform for realizing temporal interfaces for metasurface‐guided waves (MGWs). Rapidly changing metasurface resonance enables frequency conversion and temporal scattering of a concurrently propagating MGW. Using analytical methods and electromagnetic simulations, free carriers are locally generated to produce frequency‐shifted infrared MGWs. Such time interfaces can be utilized to generate large, highly localized quasistatic magnetic fields within the metasurfaces. The resulting nanoscale magnetization, supported by the residual circulating currents, persists for several optical cycles after the departure of the time‐scattered MGWs. During the rectification process, the initial electromagnetic energy of the injected MGWs is partitioned between the temporally scattered MGWs, the residual motion of the free carriers, and a quasistatic magnetic field.

## Graphical abstract

Infrared metasurface‐guided waves (IR‐MGW) scattered by a time interface (TI) induced by rapid, localized free‐carrier generation can be efficiently rectified to generate strong, persistent magnetic fields. We develop a rigorous, theoretical model to study a time‐varying, dispersive medium and uncover the redistribution of the initial energy of the IR‐MGW into a frequency‐shifted MGW and, remarkably, reveal quasistatic electronic currents that support giant nanoscale magnetization.

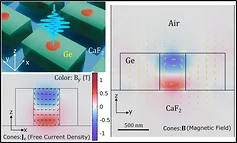

## 1 Introduction

Time‐varying media have been widely studied in plasma physics for several decades. The theory of frequency upshifting [1, 2] and spectral broadening [3] of an electromagnetic pulse was originally developed for gaseous plasmas. Rapid free‐carrier (FC) generation by a laser pulse via photoionization upshifts the pulse's frequency [4, 5]. Recent research has focused on time‐varying optical media, offering a new platform for studying phenomena such as photon acceleration (PA) [6, 7], wave amplification in momentum bandgaps using photonic time crystals [8, 9], negative wave extinction [10], phase conjugation and negative refraction [11], and surface wave control using time interfaces (TIs) [12, 13], among several other explorations of novel physical phenomena [14, 15, 16, 17, 18]. We refer the reader to the review articles in Refs. [19, 20] for a comprehensive overview of time‐varying photonics.

One of the many interesting applications of time‐varying photonics is on‐demand up‐ and down‐conversion of the incident frequency, with potential uses in optical signal processing and communications. Lately, the large optical near‐field amplification observed in doped transparent conducting oxides (TCOs) has been used to enhance light‐matter interactions [21, 22]. Intense pump pulse incidence on a TCO leads to temperature modulation of the electrons in the conduction band, which in turn affects the plasma frequency $\omega_{p}$, and has allowed for tunable redshifting of the probe pulse [7, 23]. Dynamic tuning of the probe frequency with selective blue‐ or red‐shifting has been observed with two‐color (UV and IR) excitation in an AZO thin film [24]. However, the need for two pump pulses at different frequencies renders this frequency‐shifting scheme complex and difficult to implement. Moreover, these approaches rely on coupling the probe wavelength to the epsilon‐near‐zero (ENZ) mode of the substrate, constraining the accessible spectral range as a function of doping and geometric parameters, which cannot be tuned dynamically during operation. Furthermore, a significant drawback of TCOs is their extreme lossy nature in the ENZ regime, where they are typically operated [21].

We propose tuning the resonance frequency of a metasurface to realize a time interface in a structured optical medium. Backed by numerical simulations and analytic perturbation theory, we demonstrate tunable red‐ and blueshifting of the metasurface resonance using localized free carrier generation by a pump laser beam in a hot spot at each meta‐atom. We employ this resonance‐shifting mechanism to create a TI for a mid‐IR (MIR) metasurface‐guided wave (MGW). The MGW is a transversely confined metasurface mode that propagates along the metasurface and does not leak into the air above or the dielectric substrate below. Local FC generation by a near‐IR (NIR) pump pulse in the meta‐atom, on a timescale comparable to the optical cycle of the MGW, shifts the metasurface resonance and creates a sharp TI. Furthermore, we investigate the energy dynamics of the propagating MGW across the TI. We find that after the TI, the electromagnetic energy of the launched MGW is divided between the electromagnetic energy of the temporally scattered MGW, the kinetic energy of the free carriers, and the magnetic energy in a quasistatic magnetic field created in the hot spot by the TI. The FCs generated in the hot spot are accelerated by the electromagnetic fields of the MGW during the TI, leading to the formation of quasistatic currents that circulate within the hot spot and persist after the TI. Consequently, the resonantly enhanced time‐varying AC magnetic field of the MGW is efficiently rectified, yielding a quasistatic magnetic field localized in the hot spot. Remarkably, even when the effect of electron‐scattering losses is taken into account, this rectified quasistatic magnetic field persists for several scattering times after the TI.

Nonlinear optical properties of certain materials have been used to generate (i) quasi‐static electric fields from time‐varying fields via electro‐optic rectification [25, 26], and (ii) light‐induced magnetization via the Inverse Faraday Effect (IFE) [27, 28]. However, the typically weak nonlinear optical properties of common optical materials constrain their applications. Moreover, the IFE typically generates instantaneous magnetic fields proportional to the pump‐pulse intensity. Recently, optical pump‐induced ferromagnetic order has also been reported in two‐dimensional materials [29], and laser‐assisted nanostructuring of magnetic materials has been used to generate structured magnetic fields [30]. Here, we propose an efficient method for rectifying propagating AC magnetic fields using a time interface created by localized FC generation in an all‐dielectric metasurface, which is agnostic to the nonlinear optical properties of the constituent materials. The rectification of AC magnetic fields from a propagating electromagnetic wave has been studied in the context of a uniform plasma [31]. However, to the best of our knowledge, this is the first proposal of the use of a TI in the rectification of an AC magnetic field to generate a persistent quasistatic magnetic field that leverages the resonant enhancement provided by a nanostructured metasurface and is dependent on the three‐dimensional optical fields of the MGW and the hot spot. Nanostructured three‐dimensional magnetic fields have potential applications in spintronics and computing [32].

The rest of the manuscript is organized as follows. An all‐dielectric metasurface (semiconductor meta‐atoms atop an infrared transparent substrate) that will be used throughout this work as a platform to demonstrate a TI is described in Section 2. A specific metasurface design supporting high‐quality factor MIR resonances and highly localized “hot spots” of the electric field intensity that can be used for localized free carrier generation (LFCG) is described in Section 2.1. Several perturbative techniques for deriving rigorous analytic expressions for the resonance‐frequency shifts due to local FC generation are described in Section 2.2, which account for the vector nature of electromagnetic fields within meta‐atoms. We demonstrate that, by controlling the density and spatial distribution of the generated carriers, either red‐ or blue‐shifting of a metasurface resonance can be achieved, with specific examples for a germanium‐based metasurface presented in Section 2.3. In Section 3.1, we detail our approach to the modeling of laser‐driven free carrier generation in a dielectric. The effects of FC generation on a dispersive Drude–Lorentz medium are described in Section 3.2, where we rigorously derive the expressions for the currents due to the newly created FCs. In Section 3.3 we derive an analytic expression for the total energy density in a time‐varying Drude–Lorentz medium, where we find that the total energy in the system remains unaffected by the FCs, but rather a re‐partitioning of the energy between the EM fields and the free carriers takes place. In Section 4.1, we demonstrate the effects of a sharp TI on a metasurface‐guided MIR mode using the pump‐probe approach via time‐domain simulations. An ultrashort high‐frequency pump pulse can produce temporal reflections via localized metallization for a lower‐frequency MIR mode while also redshifting the mode. We study the energy dynamics of the MGW mode across a time interface in Section 4.2 and describe the partitioning of the MGW's energy after the TI. In Section 4.3 we describe a quasistatic magnetic field that is generated as a result of the TI via rectification of the AC magnetic fields of the propagating MGW. Our findings are summarized in Section 5.

## 2 Controlling MS Optical Properties via FC Generation

The most intuitive and technically straightforward approach to rapidly modulating the refractive index of a solid material is to use a laser beam to generate high carrier densities. The process of free carrier generation can be further enhanced by using resonant dielectric or semiconductor metasurfaces. Nonlinear light‐matter interactions inside such metasurfaces are significantly enhanced by the combination of the local field enhancement at the “hot spots” with a high‐quality factor (Q) resonance that traps light for long time periods [6, 33]. Moreover, dielectric metasurfaces have significantly higher optical damage thresholds compared to their plasmonic counterparts, which suffer higher ohmic losses [34, 35] and have higher local field enhancement due to the plasmonic resonance, which would, in turn, render them more susceptible to damage. Thus, an all‐dielectric high‐Q metasurface with a tunable resonance via localized free‐electron generation by an intense pump pulse and the probe experiences the resulting change of the metasurface properties [36]. In the following sections, we introduce a metasurface‐based approach that uses spatially localized free‐carrier generation to tune the metasurface resonance frequency.

### 2.1 Metasurface Design

The metasurface design comprises a two‐dimensional array of high‐index rectangular semiconductor blocks (the meta‐atoms), unequally spaced by $p_{x(y)}$ in the x(y) directions on an optically transparent substrate, as shown in Figure 1. Note that even though the meta‐atoms will be assumed to be made of the semiconductor material (e.g., germanium) for practical purposes, they will still be referred to as “all‐dielectric metasurfaces” because of the nearly negligible frequency dependence of the refractive index of the constituent. We chose germanium because it offers near‐zero loss and near‐unity transmission in the mid‐IR regime [37] and has an extremely high laser‐induced damage threshold (∼ 2.9 $\mathrm{TW}/{\mathrm{cm}}^{2}$ for femtosecond pulses at ∼2μm [39]). The design parameters of the metasurface are detailed in the caption of Figure 1. The refractive indices of the substrate and meta‐atom material (${\mathrm{CaF}}_{2}$ and Ge, respectively) are assumed to be frequency independent for the range of mid‐infrared (MIR) frequencies of the probe.

**FIGURE 1:**
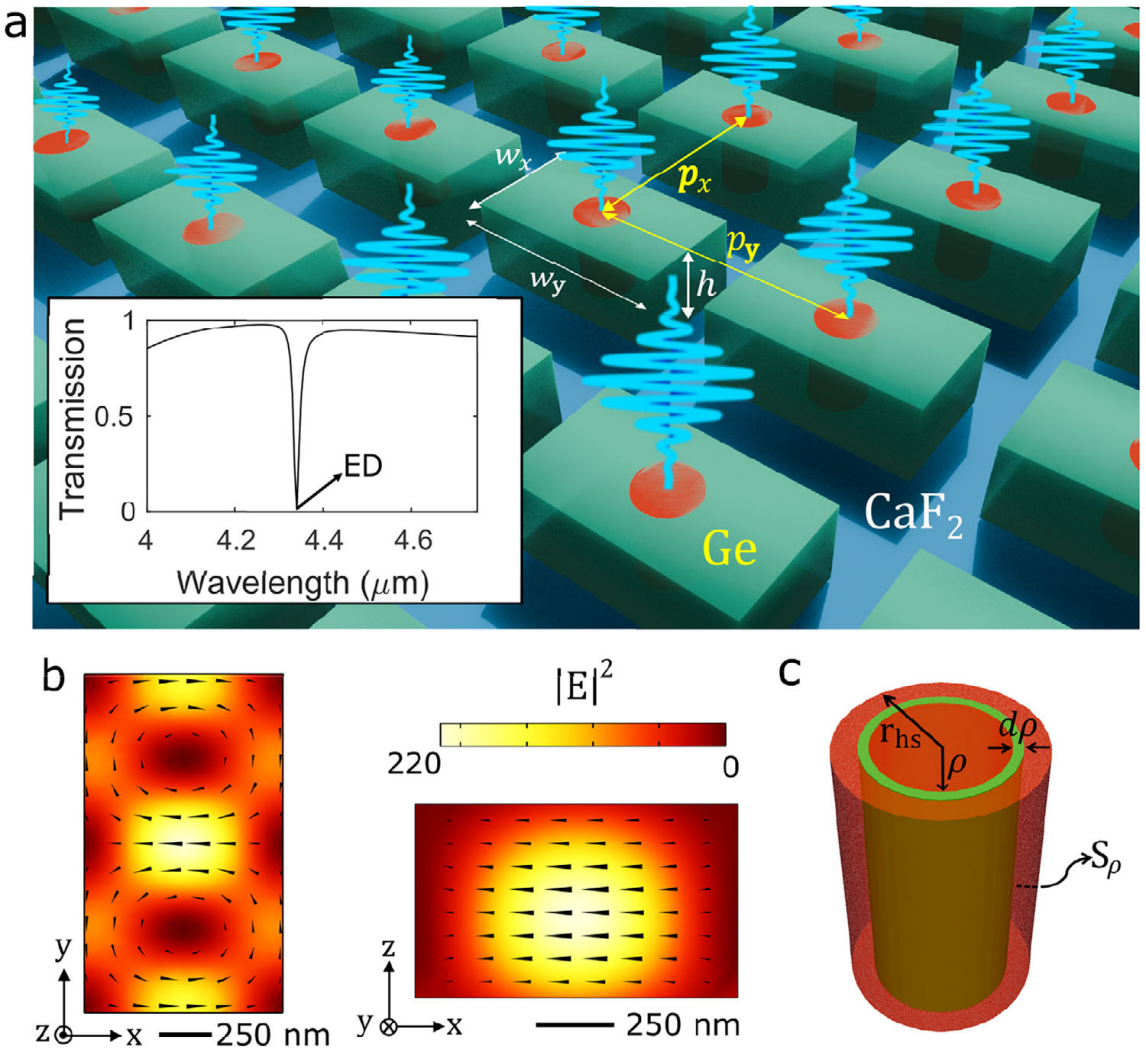
(a) Schematic of a metasurface comprising rectangular semiconductor blocks periodically arranged on an infrared‐transparent substrate. Red spots: cylindrical region where a pump pulse generates free‐carriers. Inset: metasurface transmission spectra for normally incident x‐polarized mid‐infrared light. (b) Electric field (arrows) and its intensity ${\left|E\right|}^{2}$ (color‐coded) distribution at the electric dipolar (ED) resonance in the x‐y (left panel) and x‐z (right panel) planes drawn through the middle of a meta‐atom. (c) Schematic for the perturbative calculation of the resonance frequency shift produced by free‐carrier generation inside the hot‐spot cylinder with radius $r_{\mathrm{hs}}$ using annular rings (shown in green). Materials: Germanium (Ge, $n_{\mathrm{Ge}}\approx 3.98$ [37]) for meta‐atoms, Calcium Fluoride (${\mathrm{CaF}}_{2}$, $n_{{\mathrm{CaF}}_{2}}\approx 1.4$ [38]) for the substrate. Geometric parameters: $p_{x}=1.35\;\mu m,\;p_{y}=2\;\mu m$, $w_{x}\times w_{y}\times h$: 1μm×1.7μm×0.6μm, and $r_{\mathrm{hs}}=210\;\mathrm{nm}$.

The optical properties of the metasurface are shown in the inset of Figure 1a, where the numerically calculated transmission spectrum is plotted for mid‐IR frequencies. The dip in the transmission spectrum corresponds to a high‐Q (Q∼287) electric dipolar resonance (marked ED at $\lambda_{\mathrm{ED}}∼4.34\;\mu m$). The electric field (direction and intensity) for the ED resonance in the meta‐atom's x‐z midplane is shown in Figure 1b. The electric classification of the resonance is based on the vectorial nature of the electric field and the non‐rotational nature of the electric field at $\lambda_{\mathrm{ED}}$ indicates an ED resonance. In addition to its narrow spectral width $\delta \omega \equiv \omega_{0}/Q$, the ED resonance has a second important characteristic: a strongly enhanced and highly localized electric field intensity, as shown in Figure 1b. As will be explained in Section 2.2, the amount of resonance frequency shift Δω produced by the generation of free carriers is proportional to the fraction of the energy of the electric field contained in the hot spot. Similarly, the effect of this frequency shift on the probe light stored inside the metasurface (i.e., the efficiency η of the TI) is proportional to the ratio of the resonance frequency shift Δω and the resonance bandwidth, i.e., η∼QΔω. Thus, both the resonant field enhancement and the narrow bandwidth of the high‐Q ED resonance are beneficial to creating an efficient TI.

### 2.2 Shifting the Resonance Frequency of Spectrally‐Selective Metasurfaces: Perturbation Theory

In this section, we develop a general formalism to calculate the resonance frequency shift of a high‐Q all‐dielectric metasurface using LFCG. LFCG reduces the dielectric permittivity of the constitutive material from $\varepsilon_{\mathrm{ini}}$ to $\varepsilon_{\mathrm{fin}}\equiv \varepsilon_{\mathrm{hs}}$ in the hot spot. Two LFCG regimes will be considered: (i) the perturbative regime corresponding to a low density of the free carriers ($|\varepsilon_{\mathrm{ini}}-\varepsilon_{\mathrm{fin}}|<\varepsilon_{\mathrm{ini}}$), and (ii) the non‐perturbative regime of local “metallization” corresponding to the high density of the free carriers ($\varepsilon_{\mathrm{fin}}\ll -\varepsilon_{\mathrm{ini}}$).

Small localized modifications in the permittivity of a material inside a resonator alter its resonance frequency, and this change in resonance frequency may be understood as a perturbative effect [40, 41, 42, 43]. Early work required knowledge of the exact perturbed electromagnetic fields (which are generally unknown) to predict the resonance shift. More recent work uses techniques borrowed from time‐independent perturbation theory [44] in quantum mechanics to predict resonance frequency shifts in perturbed electromagnetic cavities [45, 46, 47, 48]. We utilize the formalism outlined in Ref. [41] and derive an equation that accounts for the full‐vector electric field within the perturbed hot spot and accurately predicts fractional changes in the resonance frequency for small perturbations of the meta‐atom.

We consider a perturbation arising from a localized change in permittivity due to LFCG inside a hot spot located inside a semiconductor meta‐atom from an initial relative permittivity $\varepsilon_{\mathrm{ini}}$ to a perturbed relative permittivity $\varepsilon_{\mathrm{fin}}\left(r\right)$, where $\varepsilon_{\mathrm{fin}}(|r|>r_{\mathrm{hs}})=\varepsilon_{\infty }$ and $\varepsilon_{\mathrm{fin}}(|r|<r_{\mathrm{hs}})=\varepsilon_{\mathrm{hs}}$ where $r_{\mathrm{hs}}$ is the radius of the cylindrical hot spot. The permittivity change inside the hot spot arises from changes in the free carrier density in the hot spot $N_{e}\left(r,t\right)$, since $\varepsilon_{\mathrm{hs}}\left(\omega \right)=\varepsilon_{\infty }-\frac{\omega_{p}^{2}}{\omega^{2}+i\gamma \omega }$, and $\omega_{p}^{2}=\frac{N_{e}e^{2}}{\varepsilon_{0}m_{e}}$. Here, $\varepsilon_{\infty }=\varepsilon_{\mathrm{ini}}\approx 16$ is the high‐frequency response of bound electrons, $\varepsilon_{0}$ is the permittivity of vacuum, $m_{e}=0.041\;m_{0}$ is the effective mass of the free carriers near the Γ point of the conduction band [49], and an arbitrary electronic scattering rate, $\gamma^{-1}=100\;\mathrm{fs}$ is assumed in the hot spot to account for various losses. Following the standard perturbation theory  [41, 43], we derived the resonance frequency shift Δω using only the unperturbed electromagnetic fields (see Section S1 for details)

(1)

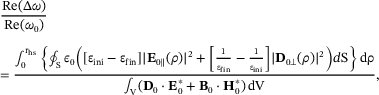

where the perturbed cylindrical hot spot comprises annular shells with radii $0<\rho <r_{\mathrm{hs}}$, thicknesses dρ, and area $S_{\rho }$ (see Figure 1c). The ⊥/∥ components of $E_{0}$/$D_{0}$ are defined with respect to the surface 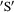 of the infinitesimally thin annular cylinder, and 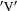 is the metasurface unit cell volume. While Equation (1) was derived for cylindrical perturbations in metasurfaces with nonmagnetic material constituents, a similar equation for calculating fractional shifts in the resonance frequency can be derived for arbitrary perturbations. Though derived for small perturbations, Equation (1) provides several key insights. First, it follows that a small reduction in the dielectric permittivity of the hot spot due to LFCG blue‐shifts the resonance frequency: Δω>0 if $0<\varepsilon_{\mathrm{fin}}<\varepsilon_{\mathrm{ini}}$. Second, we note that the blueshift Δω is expected to be particularly large when $\varepsilon_{\mathrm{fin}}\approx 0$, i.e., when the hot spot material enters the epsilon‐near‐zero (ENZ) regime. Third, Equation (1) indicates that the sign of Δω changes and the resonance redshifts when the hot spot becomes plasmonic ($\varepsilon_{\mathrm{fin}}<0$), including the regime of complete metallization ($\varepsilon_{\mathrm{fin}}\ll -\varepsilon_{\mathrm{ini}}$). The comparison between the predictions from Equation (1) and those of other methods is shown in Section S1.

Although the regime of complete metallization constitutes a strong change in the material permittivity inside the hot spot from that of the rest of the meta‐atom, we may still predict the resonance frequency shift. In fact, the calculation of the resonance frequency shift of a perturbed microwave cavity by Slater  [50] assumed the introduction of a small volume ΔV of a perfect electric conductor (PEC) into the resonator and made specific predictions about blue‐ or red‐shifts of the metasurface resonance. A detailed study of the effects of PEC introduction or localized metallization in the meta‐atom is presented in Section S2. Throughout the manuscript, we treat a PEC and complete metallization of the material inside the hot spot as interchangeable terms.

### 2.3 Example: Resonance Frequency Shifting in Ge Metasurface

Modification of the local permittivity in the Ge block provides a mechanism to tune the metasurface resonance frequency. LFCG can modify the permittivity at the hot spot (red cylinders in Ge blocks in Figure 1a). Specifically, we consider a hot spot with radius $r_{\mathrm{hs}}=210$ nm, where the local FC density varies rapidly. The value of $r_{\mathrm{hs}}$ is chosen based on the spatial profile of the FCs generated via tunneling photoionization due to a pump pulse. Tunneling ionization is a nonlinear process and therefore enables the creation of a hotspot narrower than the diffraction‐limited beam spot for a near‐IR pump (see Section S5 for details). An ultra‐short, high‐intensity pump pulse can be converted into a spatial array of focused pulses using a diffractive beam splitter [51], thereby generating hot spots in the meta‐atoms. Alternatively, the hot spots may be created by local‐field enhancement using the mid‐IR probe itself. Deep subwavelength spatial localization of FC generation has been achieved using mid‐IR pulses in the tunneling regime (e.g., the radius of the hot spot $r_{\mathrm{hs}}<\lambda /40$) [33]. In the rest of the manuscript, we shall focus on the pump‐probe method using a near‐IR pump for LFCG and a mid‐IR probe.

The evolution of the metasurface transmission spectrum (from COMSOL simulations) as the hot spot's FC density $N_{e}\left(r,t\right)$ increases from $10^{18}\;{\mathrm{cm}}^{-3}$ to $10^{22}\;{\mathrm{cm}}^{-3}$ is shown in Figure 2a. For 4μm≤λ≤5.5μm, the permittivity in the hot spot, $\varepsilon_{\mathrm{hs}}$, varies in the range $0<\varepsilon_{\mathrm{fin}}=\varepsilon_{\mathrm{hs}}<\varepsilon_{\mathrm{ini}}$ when $N_{e}\leq 10^{20}\;{\mathrm{cm}}^{-3}$. We see in Figure 2a that the dip in transmission corresponding to the metasurface ED resonance blue‐shifts as $N_{e}$ increases from $10^{18}$ to $10^{20}\;{\mathrm{cm}}^{-3}$ and $\varepsilon_{\mathrm{fin}}=\varepsilon_{\mathrm{hs}}$ decreases from $\varepsilon_{\mathrm{ini}}$ to 0 (in agreement with the prediction of Equation 1). For $N_{e}>10^{20}\;{\mathrm{cm}}^{-3}$, the ED resonance disappears and then reappears at much higher wavelengths, exhibiting a strong redshift. This is because for large FC density, the hot spot metallizes, strongly modifying the resonant fields. Even though such modifications are beyond the perturbative regime, Equation (1) can still be used to qualitatively predict the measured redshifts. When $\varepsilon_{\mathrm{hs}}=\varepsilon_{\mathrm{fin}}\to 0^{-}$ (i.e., $N_{e}\approx 10^{20+}\;{\mathrm{cm}}^{-3}$), the second term in the numerator of Equation 1 diverges, indicating a strong redshift of the metasurface resonance. However, upon further increasing the carrier density such that $N_{e}>10^{20}\;{\mathrm{cm}}^{-3}$, the resonance is still redshifted, but the magnitude of redshift decreases as the two terms in the numerator of Equation (1) compete.

**FIGURE 2:**
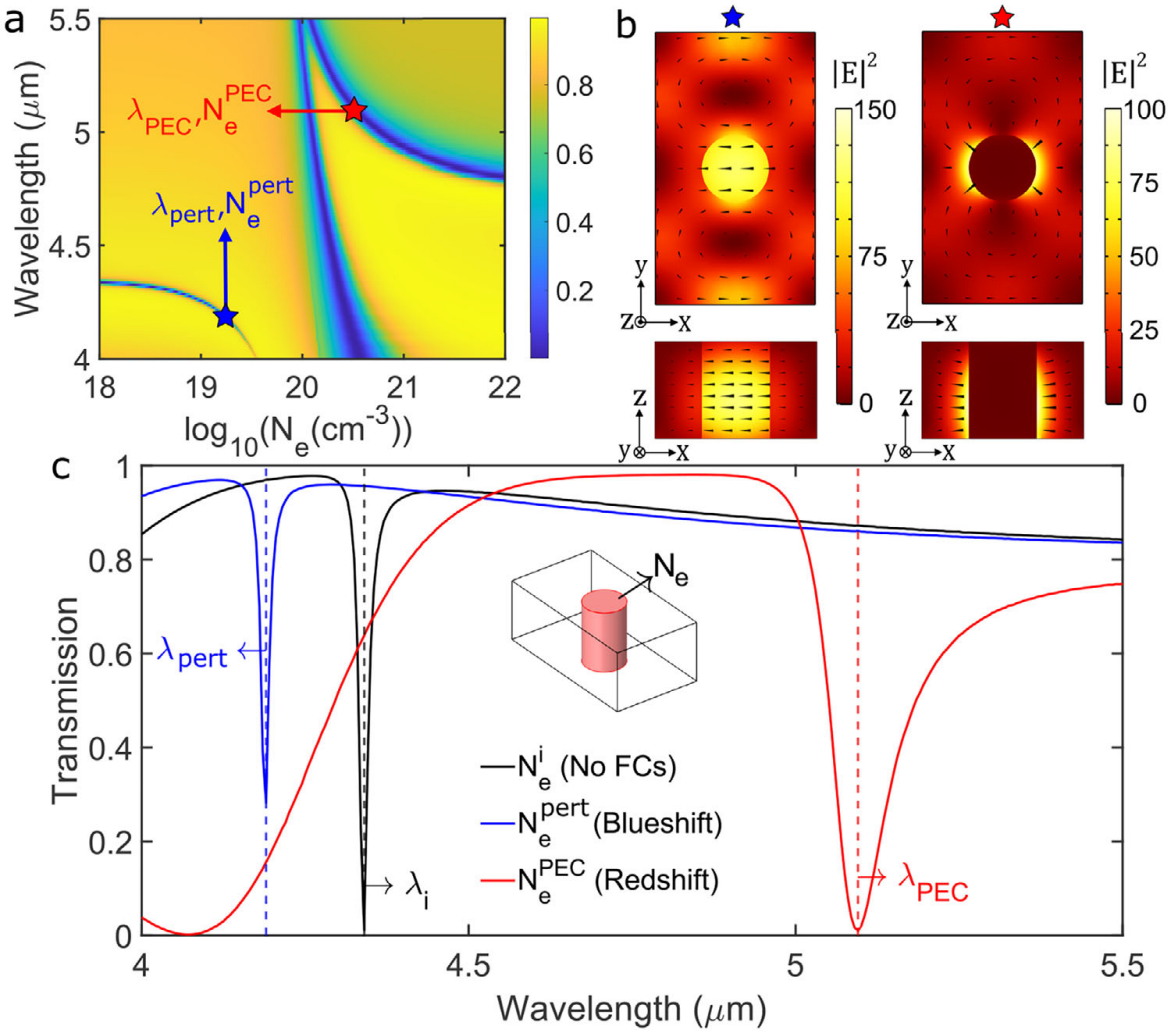
(a) Metasurface transmission as a function of hot spot free carrier density ($N_{e}\left(r,t\right)$) for normally incident x‐polarized light. (b) Intensity ${\left|E\right|}^{2}$ enhancement (color), and E (black cones) of the ED resonance in the meta‐atom's x‐y and x‐z mid‐planes for $\lambda_{\mathrm{pert}}=4.19\;\mu m$, $N_{e}^{\mathrm{pert}}=1.6\times 10^{19}\;{\mathrm{cm}}^{-3}$ (blue star) and $\lambda_{\mathrm{PEC}}=5.09\;\mu m$, $N_{e}^{\mathrm{PEC}}=3.2\times 10^{20}\;{\mathrm{cm}}^{-3}$ (red star). The stars serve as guides to the eye. (c) The black curve shows the metasurface transmission spectrum before FC generation ($N_{e}^{i}=0\;{\mathrm{cm}}^{-3}$), while the blue (red) curve shows the spectrum when the carrier density is increased to $N_{e}^{\mathrm{pert}}$ ($N_{e}^{\mathrm{PEC}}$); the metasurface parameters are the same as described in Figure 1.

The field enhancement in intensity ${\left|E\right|}^{2}$ due to the excitation of the ED resonance in the Ge block is shown in Figure 2b for two representative cases. In the first case, marked by a blue star in Figure 2a,b, we are in the perturbative regime with $0<\varepsilon_{\mathrm{hs}}<\varepsilon_{\mathrm{ini}}$. Figure 2b shows the intensity profile of the ED resonance blueshifted to $\lambda_{\mathrm{pert}}=4.19\;\mu m$ when $\;N_{e}^{\mathrm{pert}}=1.6\times 10^{19}\;{\mathrm{cm}}^{-3}$. We choose these parameters to illustrate the perturbative case, since $\varepsilon_{\mathrm{hs}}\left(\lambda_{\mathrm{pert}},N_{e}^{\mathrm{pert}}\right)∼10<\varepsilon_{\infty }\approx 16$. The spatial profile of ${\left|E\right|}^{2}$ is quite similar to the unperturbed case when $\varepsilon_{\mathrm{hs}}=\varepsilon_{\mathrm{ini}}$ (Figure 1b). The second case, marked by a red star in Figure 2a,b, lies beyond the perturbative regime where $\varepsilon_{\mathrm{hs}}\ll 0$ and the hot spot effectively behaves like a metal. We choose the resonance at $\lambda_{\mathrm{PEC}}=5.09\;\mu m$ when $N_{e}^{\mathrm{PEC}}=3.2\times 10^{20}\;{\mathrm{cm}}^{-3}$ and refer to this as the PEC case ($\varepsilon_{\mathrm{hs}}\left(\lambda_{\mathrm{PEC}},N_{e}^{\mathrm{PEC}}\right)∼-165$). In this case, we find that the electric field has been expelled from the hot spot and the spatial profile of ${\left|E\right|}^{2}$ is strongly modified. However, the spatial profile across the rest of the Ge block indicates that the same ED resonance has been excited. The transmission spectra of the metasurface for three different values of the FC density at the hot spot $N_{e}\left(r,t\right)$ are shown in Figure 2c. The black curve shows the metasurface spectrum for the unperturbed case before the pump generates any FC ($\varepsilon_{\mathrm{hs}}=\varepsilon_{\mathrm{ini}}$), with the ED resonance at $\lambda_{i}=4.34\;\mu m$. The blue curve shows the spectrum for $N_{e}=N_{e}^{\mathrm{pert}}$, where we see significant blueshifting of the ED resonance. The resonance blueshifts to $\lambda_{\mathrm{pert}}=4.19\;\mu m$, and the Q factor increases to $Q_{\mathrm{pert}}=318$. However, upon further increasing $N_{e}$ while maintaining $\varepsilon_{\mathrm{hs}}>0$, the quality factor of the resonance decreases, and it vanishes completely in the regime where $\varepsilon_{\mathrm{hs}}=0$. The red curve shows the spectrum for $N_{e}=N_{e}^{\mathrm{PEC}}$ when the hot spot is metalized and the resonance redshifts significantly to $\lambda_{\mathrm{PEC}}=5.09\;\mu m$ with $Q_{\mathrm{PEC}}=53$.

An electrostatic model that calculates the effective permittivity of the meta‐atom for variable free‐electron densities within the hot spot is presented in Section S3. The model qualitatively predicts the shifts in the metasurface resonance frequency when the hot spot's relative permittivity is varied between $-\infty <\varepsilon_{\mathrm{hs}}\leq \varepsilon_{\infty }$ by calculating the effective capacitance and hence the effective permittivity due to the hot spot inclusion ($\varepsilon_{\mathrm{hs}}\left(N_{e}\left(r,t\right)\right)$) inside a capacitive meta‐atom when a constant potential difference is applied across the meta‐atom.

## 3 Modeling Time Interfaces in Semiconductors Using Free Carrier Generation

Locally generating FCs within the nanostructures of a metasurface enables dynamic tuning of the effective permittivity and resonance frequency of the metasurface. This makes LFCG in an all‐dielectric metasurface a promising platform for time‐varying photonics at mid‐IR wavelengths. To understand the optical effects of LFCG in the hot spot, we modeled each meta‐atom as a time‐varying Drude–Lorentz medium and derived the wave equation for the time‐varying metasurface. Specifically, we study the impact of resonance shifting via controlled LFCG in the hot spot to theoretically demonstrate that the metasurface resonance can be tuned on the order of an optical cycle of the mid‐IR probe (i.e., the metasurface‐guided wave, or MGW) by an optical pump pulse, thereby realizing a sharp TI. In subsequent sections, we study in detail the case in which LFCG induces a redshift in the mid‐IR probe MGW.

### 3.1 Laser‐Induced Free Carrier Generation

Upon irradiation with an intense laser pulse, FCs can be generated via photoionization. We use the Keldysh model [52] to calculate the photoionization rate $R_{\mathrm{PI}}$ and obtain the FC density ($N_{e}$) in Ge as

(2) $$\frac{\partial N_{e}}{\partial t}=R_{\mathrm{PI}}\left(I[t\right])-\frac{N_{e}}{\tau_{e-h}}$$

where $\tau_{e-h}$ is the electron‐hole recombination time, and the partial time derivative indicates that $N_{e}$ has both spatial and time dependence. We use a pump pulse centered at $\lambda_{p}=1580\;\mathrm{nm}$, with pulse width $\tau_{p}=$ 8 fs, focused at the hot spot for LFCG, which modifies $\varepsilon_{\mathrm{hs}}$. Near‐IR ultrafast pulses have been experimentally realized [53], and are essential for rapid FC generation to produce a sharp TI. The pump intensity incident at the hot spot is $I\left[t\right]=2I_{\mathrm{avg}}\cos^{2}\left(k_{p}\left(z+\mathrm{ct}\right)\right)$ $e^{-2{\left(t-\tau_{1}\right)}^{2}/\tau_{p}^{2}}$, where $k_{p}=2\pi /\lambda_{p}$, and we choose $I_{\mathrm{avg}}=2.075$ $\mathrm{TW}/{\mathrm{cm}}^{2}$ and $\tau_{1}=3\;\mathrm{ps}$. $\tau_{e-h}$ in Ge is on the order of hundreds of picoseconds and thus is much longer than the time period and pulse width of the optical pump and the mid‐IR MGWs [54]. For the time scales of interest for a single sharp time interface, where $N_{e}$ increases over a few fs, the effect of $\tau_{e-h}$ on $N_{e}$ is negligible. However, the choice of a material with shorter relaxation times may play a critical role in future studies aimed at creating time slabs or time crystals. Therefore, the FC population in the hot spot is maintained for a sufficiently long time, and we restrict our analysis to the sharp TI between the two phases of the hot spot, i.e., the unionized phase (before the TI) and the ionized phase (after the TI). Further details on the calculation of $N_{e}$ using the Keldysh model are provided in Section S4. The Keldysh model for photoionization by an optical field accounts for both the tunneling ionization and the multiphoton absorption. For the material comprising the metasurface, Ge (band gap, Δ≈0.8 eV, and electron's reduced mass, $m_{e}=0.041\;m_{0}$) [49], and the given pump pulse parameters, the Keldysh parameter $\gamma_{p}=k_{p}c\sqrt{m_{e}\Delta }/\mathrm{eF}\approx 0.547$ (average electric field strength $F=\sqrt{2I_{\mathrm{avg}}/c\varepsilon_{0}n_{p}^{2}}$, $n_{p}=4.2$ is the Ge refractive index at the pump wavelength) [37, 52]. Since $\gamma_{p}\ll 1$, tunneling ionization is the dominant mechanism [55] and the newly formed free electrons possess negligible kinetic energy.

### 3.2 Time‐Varying Dispersive Medium

We model Ge as a Drude–Lorentz dispersive medium to understand the optical effects of FC generation at the hot spot by an intense pump pulse. The displacement field D and the polarization current $J_{e}$ in a Drude–Lorentz medium are $D=\varepsilon_{0}\varepsilon_{\infty }E+P_{e}$, where, $J_{e}=\partial_{t}P_{e}$ where $P_{e}$ is the density of the FC‐induced dipole moment. The polarization current, $J_{e}$, is formed as a result of the interaction of MGW fields with FCs. We consider a medium with pre‐existing free electron density $N_{e}^{\left(1\right)}$ and a time‐dependent density of electrons created by tunneling ionization $N_{e}^{\left(2\right)}\left(t\right)$ such that the total FC density in the medium is $N_{e}\left(t\right)=N_{e}^{\left(1\right)}+N_{e}^{\left(2\right)}\left(t\right)$. Under the two‐fluid approximation for two different electronic populations, where we consider only the electronic motion in a neutral plasma, the polarization current density is given by, $J_{e}\left(t\right)=J_{e}^{\left(1\right)}\left(t\right)+J_{e}^{\left(2\right)}\left(t\right)=-{\mathrm{eN}}_{e}^{\left(1\right)}v_{e}^{\left(1\right)}\left(t\right)-e\int_{-\infty }^{t}\partial_{t^{\prime }}N_{e}^{\left(2\right)}v_{e}^{\left(2\right)}\left(t,t^{\prime }\right){\mathrm{dt}}^{\prime }$, where $J_{e}^{\left(1\right)}$ and $J_{e}^{\left(2\right)}$ are the electron current densities due to the pre‐existing and newly created free electrons, respectively. $v_{e}^{\left(1\right)}\left(t\right)$ is the velocity of a pre‐existing electron, and $v_{e}^{\left(2\right)}\left(t,t^{\prime }\right)$ is the velocity at time t of an electron created at time $t^{\prime }$ (where $t>t^{\prime }$) [56]. These velocities, due to acceleration by the electric field of an electromagnetic wave, are calculated as $v_{e}^{\left(1\right)}\left(t\right)=-\left(e/m_{e}\right)\int_{-\infty }^{t}E\left(t^{\prime \prime }\right){\mathrm{dt}}^{\prime \prime }=\left(e/m_{e}\right)A\left(t\right),\;v_{e}^{\left(2\right)}\left(t,t^{\prime }\right)=-\left(e/m_{e}\right)\int_{t^{\prime }}^{t}E\left(t^{\prime \prime }\right){\mathrm{dt}}^{\prime \prime }=\left(e/m_{e}\right)\left[A\left(t\right)-A\left(t^{\prime }\right)\right]$, where A is the magnetic vector potential, and we have used $E=-\partial_{t}A$. Note that when calculating $J_{e}^{\left(2\right)}$, we only consider the acceleration of the FCs after their creation at time $t^{\prime }$. Here we neglect the non‐parabolicity of the electronic bands and assume the same effective mass $m_{e}$ for all electrons in the conduction band of Ge. The sum of the two polarization current densities due to the pre‐existing carriers and the new carriers generated by LFCG due to the pump pulse is given as

(3) $$\begin{array}{ccc} J_{e}^{\left(1\right)}+J_{e}^{\left(2\right)} & = & \frac{\partial P_{e}}{\partial t}=-\frac{e^{2}N_{e}^{\left(1\right)}}{m_{e}}A\left(t\right)-\frac{e^{2}N_{e}^{\left(2\right)}\left(t\right)}{m_{e}}A\left(t\right)\\ & & +\frac{e^{2}}{m_{e}}\int_{-\infty }^{t}\frac{\partial N_{e}^{\left(2\right)}}{\partial t^{\prime }}A\left(t^{\prime }\right){\mathrm{dt}}^{\prime } \end{array}$$

Calculating the first‐order time derivative of $J_{e}$ from Equation (3), yields

(4) $$\begin{array}{c} \frac{\partial J_{e}}{\partial t}=\frac{\partial^{2}P_{e}}{\partial t^{2}}=\frac{e^{2}}{m_{e}}\left[N_{e}^{\left(1\right)}+N_{e}^{\left(2\right)}\left(t\right)\right]E=\frac{e^{2}N_{e}\left(t\right)}{m_{e}}E=\varepsilon_{0}\omega_{p}^{2}\left(t\right)E \end{array}$$

Here, we emphasize that $\partial_{t}J_{e}$ is independent of $\partial_{t}N_{e}$. This implies that the free electrons created at time “t” do not contribute to the current density at the same instant because the electrons are created at rest. Now, starting from Ampere's law, we formulate the wave equation in terms of A as

(5) $$\nabla \times \left(\nabla \times A\right)+\frac{\varepsilon_{\infty }}{c^{2}}\frac{\partial^{2}A}{\partial t^{2}}-\mu_{0}\frac{\partial P_{e}}{\partial t}=0;\;H=\frac{1}{\mu_{0}}\left(\nabla \times A\right)$$

COMSOL's time‐domain solver uses Equation (5) in combination with Equation (4) to simulate the interaction between an MGW and FCs at the hot spot. We numerically solve Equation (2) to obtain the temporal evolution of $N_{e}$ for a pump pulse and use it to model a sharp TI for the MGW. During the TI, FCs created at rest are accelerated by the electromagnetic field, thereby generating DC currents in the system. These constant currents establish a quasistatic magnetic field ($H_{s}$) and consequently a quasistatic magnetic vector potential ($A_{s}$) in the system. The quasistatic magnetic field (QS) mode is a zero‐frequency mode that is temporally invariant but spatially varying. After the TI ($t>t_{\mathrm{TI}}$), under the quasi‐monochromatic wave approximation, we can write the total magnetic vector potential as $A\left(r,t\right)=A_{s}\left(r\right)+A_{t}\left(r,t\right)+c.c.$, where $A_{t}\left(r,t\right)=A_{t}0\left(r\right)e^{-i\omega_{f}t}$ denotes the amplitude of the time‐varying or AC component of the magnetic vector potential and $\omega_{f}$ is the frequency of the EM wave after TI. In general, for $t>t_{\mathrm{TI}}$, using the ansatz above for A(r,t) in Equations (3) and (5), and taking only the time‐independent components (see Section S7), the amplitude of the QS mode at the hot spot A(r) satisfies

(6) $$\begin{array}{ccc} H_{s}\left(r\right) & = & \frac{1}{\mu_{0}}\nabla \times A_{s}\left(r\right);\;\;\left(\omega_{p}^{2}-c^{2}\nabla^{2}\right)A_{s}\left(r\right)\\ & = & \frac{e^{2}}{\varepsilon_{0}m_{e}}\int_{-\infty }^{t}\frac{\partial N_{e}^{\left(2\right)}}{\partial t^{\prime }}A\left(r,t^{\prime }\right){\mathrm{dt}}^{\prime } \end{array}$$

The net radiation from the QS mode is zero ($E_{s}=-\partial_{t}A_{s}=0$). This nonradiative zero‐frequency mode consumes a finite fraction of the total energy of the MGW, which is stored in the FCs' DC motion. In the next section, we study the energy dynamics in a time‐varying Drude–Lorentz medium.

### 3.3 Energy Relations

In this section, starting from Poynting's theorem, we derive an analytic expression for the total energy density in a time‐varying Drude–Lorentz medium. We then numerically demonstrate that the total energy of a dispersive system is conserved across a TI. During simulations, electromagnetic radiation escapes the simulation domain through the scattering boundaries. From Poynting's theorem, the rate of change of the total energy density can be written as (see Section S6), $\partial_{t}U=-\nabla \cdot \left(E\times H\right)=0.5*\partial_{t}\left(\varepsilon_{0}\varepsilon_{\infty }{\left|E\right|}^{2}+\mu_{0}{\left|H\right|}^{2}\right)+E\cdot \partial_{t}P_{e}$. Using $A\left(r,t\right)=A_{s}\left(r\right)+A_{t}\left(r,t\right)+c.c.$ in conjunction with the expressions for current given in Equation (3), we can separate the time‐dependent electromagnetic energy density terms and the quasistatic current driven DC terms for $t>t_{\mathrm{TI}}$. The electromagnetic energy density is $U_{\mathrm{EM}}=0.5*(\varepsilon_{0}\varepsilon_{\infty }{\left|E\right|}^{2}+\mu_{0}{\left|H\right|}^{2}+\varepsilon_{0}\omega_{p}^{2}\left(t\right)|A_{t}{|}^{2})$ and the residual or DC in the system due to the QS mode is $J_{e,s}=-\left(e^{2}N_{e}\left(t\right)/m_{e}\right)A_{s}\left(r\right)+\left(e^{2}/m_{e}\right)\int_{-\infty }^{t}\partial_{t^{\prime }}N_{e}^{\left(2\right)}A\left(r,t^{\prime }\right){\mathrm{dt}}^{\prime }$. Then, using these terms, we obtain $-\partial_{t}U_{\mathrm{EM}}=\nabla \cdot \left(E\times H\right)+E\cdot J_{e,s}$. We observe that the rate of change of $U_{\mathrm{EM}}$ after the TI is equal to the sum of the electromagnetic flux leaving the system and the rate of work done on the static currents created in the system during a TI. We emphasize that $J_{e,s}$ is a constant current density related to the DC motion of the FCs that is created during a TI and persists even after the departure of the electromagnetic waves. Finally, integrating the equation for total energy (see Section S6 for detailed derivations), yields

(7) $$\begin{array}{c} \begin{array}{c} U=\frac{1}{2}\varepsilon_{0}|E{|}^{2}+\frac{1}{2}\mu_{0}|H{|}^{2}+\frac{1}{2}\varepsilon_{0}\left(\varepsilon_{\infty }-1\right)\left|E{|}^{2}+\frac{1}{2}\varepsilon_{0}\omega_{p}^{2}\right|A{|}^{2}\\ +\int_{-\infty }^{t}\frac{\partial N_{e}^{\left(2\right)}}{\partial t^{\prime }}\frac{|eA\left(t^{\prime }\right){|}^{2}}{2m_{e}}{\mathrm{dt}}^{\prime }-A\left(t\right)\cdot \left(\frac{e^{2}}{m_{e}}\int_{-\infty }^{t}\frac{\partial N_{e}^{\left(2\right)}}{\partial t^{\prime }}A\left(t^{\prime }\right){\mathrm{dt}}^{\prime }\right) \end{array}\;\;\; \end{array}$$

The expression above for the total energy density holds at all times, including the time before, during, and after the time interface. The first (second) term on the RHS of Equation (7) represents the energy density stored in the form of electric (magnetic) fields. In contrast, the third term represents the potential energy density stored in the bound electrons. The sum of the first three terms is labeled $U_{\mathrm{Field}}$, while the sum of the remaining terms represents the energy density stored in the FCs and is labeled $U_{\mathrm{Carrier}}$. In the next section, we use Equation (7) to calculate and compare the total energies for cases with and without a TI.

## 4 Impact of a Time Interface in a Metasurface

### 4.1 Temporal Scattering of MGWs

The TI is created by FC generation in the hot spot under intense pump‐pulse illumination, resulting in a time‐varying metasurface. A MGW propagating along the metasurface is temporally scattered by the TI created by this time‐varying metasurface. Metasurface eigenmode and TI simulations are performed using COMSOL Multiphysics. The metasurface dispersion plot for the ED mode is shown in Figure 3a for the unperturbed (black line) and perturbed (red line) cases. Although the ED mode of the unperturbed metasurface leaks into the ${\mathrm{CaF}}_{2}$ substrate for most of the Brillouin zone, it drops below the light line at high $k_{x}$. It becomes an ED MGW (decaying in the z direction away from the metasurface) near the edge of the Brillouin zone ($|k_{x}|⪆0.89\cdot \pi /p_{x}$). We excite an ED MGW, propagating in the x direction with $k_{x}=0.92\pi /p_{x}$, $\;k_{y}=k_{z}=0$ (dashed gray line in Figure 3a) $\varepsilon_{\mathrm{hs}}=\varepsilon_{\mathrm{ini}}$. The MGW has a larger wave vector than the free‐space light, $|k_{x}|>2\pi /\lambda_{0}$, where $\lambda_{0}=4.27\;\mu m$ is the central wavelength of the mid‐IR probe, which excites the ED MGW. Thus, we used an array of phased electric dipoles to excite the MGW. These dipoles, as well as the simulation domain, are shown in Figure S5a. The dipole moment of the n‐th dipole (at $x_{n}=nd_{x},\;n$ = 1,2,3,...) at time t, $p_{\mathrm{dipole}}\left(x_{n},t\right)=p_{0}\cos\left(k_{x}x_{n}-\omega_{0}t\right)e^{-{\left(t-\tau_{2}\right)}^{2}/\tau_{0}^{2}}\hat{z}$ (where $\omega_{0}=2\pi c/\lambda_{0}$, $p_{0}=1$ C‐m $\tau_{2}=300$ fs and $\tau_{0}=200$ fs). The green‐shaded region in Figure 3a shows the spectrum of the dipolar excitation. The phased dipole array is essential to efficiently excite the MGW at a specific $k_{x}$, as the dispersion line for the ED MGW is extremely flat.

**FIGURE 3:**
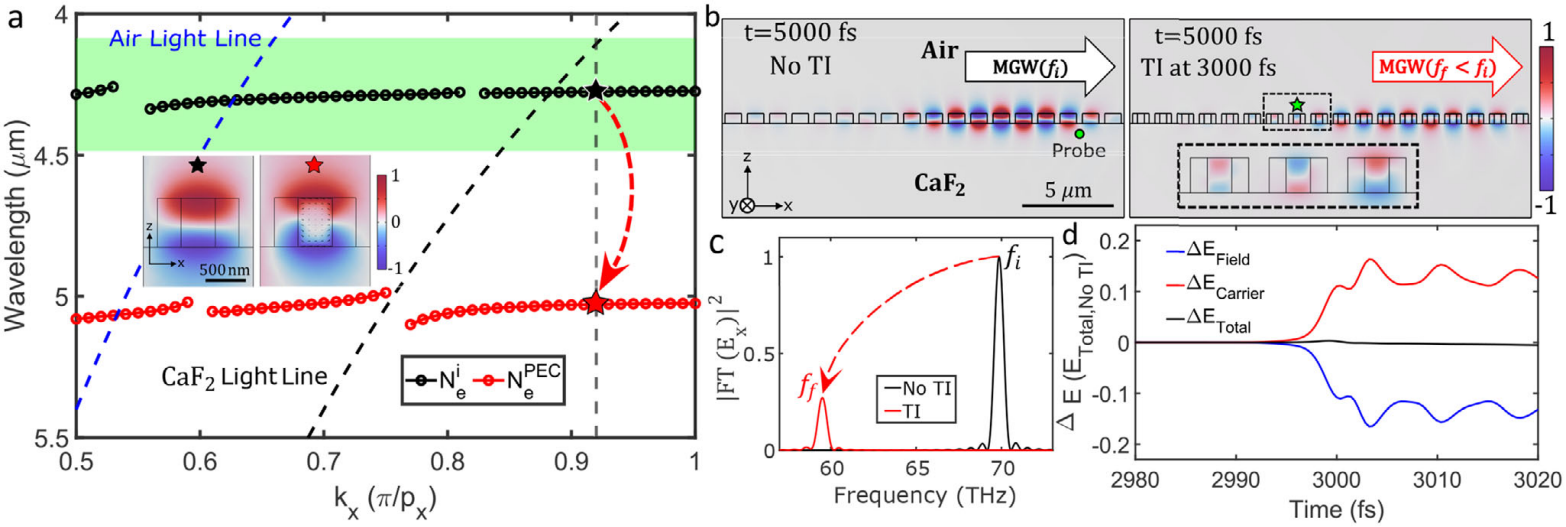
(a) Dispersion plot of the ED mode; green shaded region indicates the excitation source (dipole) spectrum. Inset: $H_{y}$ field profile of the ED mode (in the x‐z midplane) for the black/red star markers. (b) Normalized $H_{y}$ profile in the x‐z mid‐plane of the simulation domain on the left (right) shows the MGW propagating with group velocity $v_{g}^{i}\approx 0.007c$ ($v_{g}^{\mathrm{PEC}}\approx 0.009c$) without a TI (with a TI) at t = 5000 fs; the rectangular region inside each meta‐atom represents the cylindrical hot spot of radius 210 nm. The light green star indicates the meta‐atom, where the magnetic field is shown in Figure 4. (c) $|\mathrm{FT}\left(E_{x}\right){|}^{2}$ recorded at the probe after the TI for the two cases: $N_{e}^{\left(2\right)}=N_{e}^{i}=0\;{\mathrm{cm}}^{-3}$ (No TI, black line), and $N_{e}^{\left(2\right)}=N_{e}^{\mathrm{PEC}}=3.2\times 10^{20}\;{\mathrm{cm}}^{-3}$ (TI, red line); TI produced by an ionizing laser (pump) pulse at $\lambda_{p}=1580\;\mathrm{nm}$ as described in Section 3.1. (d) Time evolution of $\Delta E_{\mathrm{Field}}$: blue line, $\Delta E_{\mathrm{Carrier}}$: red line, $\mathrm{and}\Delta E_{\mathrm{Total}}$: black line; the metasurface parameters are the same as described in Figure 1.

Alterations in the constitutive materials of the metasurface over time (e.g., via controlled LFCG at the hot spot by a pump pulse) modify the metasurface, resulting in a time‐varying medium for the MGW. For the pump pulse parameters given in Section 3.1, we calculated the FC concentration in the hot spot using Equation (2) and the transition time in which $N_{e}$ changes from $N_{e}^{i}$ to $N_{e}^{\mathrm{PEC}}$ is $T_{\mathrm{trans}}\approx \;16\;\mathrm{fs}≳T_{0}=2\pi /\omega_{0}\approx 14.27$ fs. The transition from $N_{e}^{i}$ to $N_{e}^{\mathrm{PEC}}$ occurs in slightly over an optical cycle of the MGW, thus creating a moderately sharp TI for the propagating MGW. We compare the $H_{y}$ field profiles at 5000 fs in the cases with and without a TI. For the case without a TI, the MGW crosses approximately 8 meta‐atom unit cells (see Figure 3b) from left to right in the simulation domain, propagating at a group velocity of $v_{g}^{i}\approx 0.007c$, where c is the speed of light in vacuum. We compare this case with that of a TI at 3000 fs. In 3000 fs, the unperturbed MGW propagates across 5 meta‐atoms. However, at 5000 fs in the case with a TI, we observe that the MGW crosses one extra meta‐atom propagating at an increased group velocity of $v_{g}^{\mathrm{PEC}}\approx 0.009c$ in comparison to the unperturbed MGW, thus crossing a total of 9 meta‐atoms. To clearly view the magnetic field rectified during the TI, we show an enlarged view of some of the meta‐atoms enclosed in a dashed black box in the right‐field plot of Figure 3b. The enclosed meta‐atoms clearly exhibit a rectified magnetic field in the hot spot region. Furthermore, we provide evidence of temporal scattering by showing that the MGW frequency redshifts across the TI [57]. The x component of the electric field ($E_{x}$) is recorded at the probe location (shown as a yellow circle in the left panel in Figure 3b). Fourier transforms of $E_{x}$ for the two cases, with a TI at 3000 fs ($|\mathrm{FT}\left(E_{\mathrm{PEC}}\right){|}^{2}$) and without a TI ($|\mathrm{FT}\left(E_{i}\right){|}^{2}$), are shown in Figure 3c. In both cases, we focus exclusively on the electric field measured after 3000 fs. For the case without a TI, we observed a peak in the spectrum (black line in Figure 3c) at 70.1 THz (≈ 4.27 μm) corresponding to the ED resonance of the metasurface. Now, in the presence of a sharp TI (red line in Figure 3c), the spectrum of the recorded field exhibits a clearly red‐shifted ED peak at ∼59.4 THz (≈ 5.05 μm). Thus, we see that the TI due to $N_{e}$ changing from $N_{e}^{i}\to N_{e}^{\mathrm{PEC}}$ results in the redshifting of the MGW by exactly the amount predicted by the eigenmode dispersions for $N_{e}^{i}$ and $N_{e}^{\mathrm{PEC}}$ FC densities (dashed red arrow in Figure 3a).

### 4.2 Energy Dynamics Across a Time Interface

We consider pre‐existing FC density $N_{e}^{\left(1\right)}=0$ for the TI simulations. We integrate the expressions for the energy densities from Equation (7) over the simulation domain to calculate the time evolution of the energy contributions of the different terms (that is, $E_{\mathrm{Field}}=\int U_{\mathrm{Field}}\mathrm{dV}$, and $E_{\mathrm{Carrier}}=\int U_{\mathrm{Carrier}}\mathrm{dV}$). We calculate the difference in $E_{\mathrm{Field}}$, $E_{\mathrm{Carrier}}$, and also the total energy of the system ($E_{\mathrm{Total}}=\int \mathrm{UdV}$) for the cases with and without a TI ($\Delta E_{\mathrm{Total}}=\Delta E_{\mathrm{Field}}+\Delta E_{\mathrm{Carrier}};$ ΔE=E(TI)−E(NoTI) and plot these in Figure 3d. We observe that when the TI is introduced, $\Delta E_{\mathrm{Field}}$ decreases, while $\Delta E_{\mathrm{Carrier}}$ increases by an equal magnitude. Note that $\Delta E_{\mathrm{Total}}$ drops slightly below the zero line after the TI. This can be attributed to the simulation domain not being a closed system, as it contains scattering boundaries. Since the group velocity increases and the quality factor of the MGW decreases after TI, the energy in the system escapes the system more rapidly through the boundaries. In any case, ignoring these small contributions to the total energy, our simulations indicate that the system's total energy in the presence of TI (created via LFCG) is the same as in its absence, i.e, U(no TI)${|}_{t>t_{\mathrm{TI}}}$ = U(TI)${|}_{t>t_{\mathrm{TI}}}$ where $t_{\mathrm{TI}}=3000\;\mathrm{fs}$. Now, for the case of no TI, we use the unperturbed fields ($E_{0}$, $H_{0}$, $\omega_{0}$) to calculate the energy density U(no TI)${|}_{t>t_{\mathrm{TI}}}$ after the TI, which, time averaged over an optical cycle, can be written as

(8) $$\begin{array}{ccc} U\left(\mathrm{no}\;\mathrm{TI}\right){|}_{t>t_{\mathrm{TI}}} & = & U_{\mathrm{EM}}\;\left(\mathrm{no}\;\mathrm{TI}\right)=\frac{1}{2}\varepsilon_{0}\varepsilon_{\infty }\langle |E_{0}{|}^{2}\rangle +\frac{1}{2}\mu_{0}\langle \left|H_{0}{|}^{2}\right\rangle \\ & & +\frac{1}{2}\varepsilon_{0}\left(\frac{\omega_{p0}^{2}}{\omega_{0}^{2}}\right)\langle \left|E_{0}{|}^{2}\right\rangle \end{array}$$

where $\omega_{p0}$ and $U_{\mathrm{EM}}\;\left(\mathrm{no}\;\mathrm{TI}\right)$ are the plasma frequency and the electromagnetic energy density for the case without TI, respectively. Here, “⟨⟩” denotes the time‐averaged quantity over an optical cycle of the wave's frequency. However, for the case with the TI, to calculate the time‐averaged total energy density, we use $A\left(r,t\right)=A_{s}\left(r\right)+A_{t}\left(r,t\right)+c.c.$ in Equation (7) to separate the energy density in propagating electromagnetic fields from the energy density in the QS mode such that $U\left(\mathrm{TI}\right){|}_{t>t_{\mathrm{TI}}}=U_{\mathrm{EM}}\;\left(\mathrm{TI}\right)+U_{\mathrm{QS}}$.

(9) $$\begin{array}{c} U_{\mathrm{EM}}\;\left(\mathrm{TI}\right)=\frac{1}{2}\varepsilon_{0}\varepsilon_{\infty }\left\langle \right|E_{t}{|}^{2}\rangle +\frac{1}{2}\mu_{0}\left\langle \right|H_{t}{|}^{2}\rangle +\frac{1}{2}\varepsilon_{0}\left(\frac{\omega_{p}^{2}}{\omega_{f}^{2}}\right)\left\langle \right|E_{t}{|}^{2}\rangle \end{array}$$

Here, $U_{\mathrm{EM}}\;\left(\mathrm{TI}\right)$ is the electromagnetic energy density for the case of TI. The energy density in the hot spot due to the QS mode of the system can be expressed using the quasistatic magnetic field ($H_{s}$) and the quasistatic magnetic vector potential ($A_{s}$) derived in Equation (6) as

(10) $$\begin{array}{ccc} U_{\mathrm{QS}} & = & \frac{1}{2}\mu_{0}|H_{s}{|}^{2}+\frac{1}{2}\varepsilon_{0}\omega_{p}^{2}{\left|A_{s}\right|}^{2}+\int_{-\infty }^{t}\frac{\partial N_{e}^{\left(2\right)}}{\partial t^{\prime }}\frac{|eA\left(t^{\prime }\right){|}^{2}}{2m_{e}}{\mathrm{dt}}^{\prime }\\ & & -A_{s}\cdot \left(\frac{e^{2}}{m_{e}}\int_{-\infty }^{t}\frac{\partial N_{e}^{\left(2\right)}}{\partial t^{\prime }}A\left(t^{\prime }\right){\mathrm{dt}}^{\prime }\right) \end{array}$$

From Equations ((8), (9), (10)), it follows that in the case of TI, the electromagnetic energy is depleted by the same amount as the energy expended to create DC currents, $U_{\mathrm{EM}}\;\left(\mathrm{TI}\right)-U_{\mathrm{EM}}\;\left(\mathrm{no}\;\mathrm{TI}\right)=\Delta U_{\mathrm{EM}}=-U_{\mathrm{QS}}$. Numerically, we find $U_{\mathrm{QS}}>0$, which implies that the electromagnetic energy density in the MGW decreases, while the energy density in the QS mode increases across the TI.

### 4.3 Nanoscale Magnetization Using Rapid FC Generation

The MGW electric field accelerates the electrons generated at rest within the hot spot during the TI. Once they acquire a non‐zero velocity, they are subject to the magnetic field of the mid‐IR MGW present in the hot spot. After the TI, the large number of FCs in the hot spot screens the MGW electric field. Consequently, the displacement currents in the hot spot volume before the TI due to the AC electric field of the MGW are replaced by real electric currents because of the motion of the FCs in the hot spot after the TI. These circulating currents within the hot spot produce a quasistatic magnetic field that is obtained by rectifying the MGW's AC magnetic field during the TI. The acceleration of the FCs that are created during the TI by the mid‐IR MGW is driven by the magnetic vector potential, A(t), of the MGW. The long wavelength mid‐IR MGW is better able to accelerate the electrons because A(t) scales linearly with the wavelength. Additionally, the energy of the longer‐wavelength photon in the mid‐IR MGW is below the Ge bandgap, thereby eliminating the possibility of single‐photon absorption and, consequently, FC generation. Another process that can generate FC via the mid‐IR MGW is multiphoton absorption. However, the multiphoton absorption probability of a mid‐IR MGW is drastically reduced because the Keldysh parameter for the MGW, which is approximately the ratio of the TI duration to the MGW optical cycle, is less than one [58]. The rectification of the magnetic field of an electromagnetic wave upon the rapid creation of a plasma around a portion of the wave was studied several decades ago [31]. More recently, the rectification of electric fields in capacitive metasurfaces due to a time interface has been reported [12]. The opposite effect has also been predicted, where a static electric field is partially converted to a dynamic radiation field at a time interface [59]. Here, we demonstrate the rectification of the resonantly enhanced magnetic field of an MGW, which is dependent on the full‐vector, three‐dimensional optical fields of the MGW in the hot spot.

#### 4.3.1 Quasistatic Magnetic Field

The rectification of the magnetic field of the MGW at the TI is shown in Figure 4a, which shows snapshots of $H_{y}$ in the y‐z mid‐plane at different times inside the meta‐atom and its vicinity in air and the substrate. The yellow cones show the direction of the magnetic field. $H_{y}$ is normalized to the peak field of steady‐state MGW before TI. The first panel of Figure 4a shows the magnetic field at 2995 fs, when some FCs are present in the hot spot. The second (third) panel shows the magnetic field of the MGW at 3000 (3005) fs, during the middle (toward the end) of the TI. Compared with the first and second panels, the third panel exhibits marked differences in the spatial structure of the magnetic field. There is a clear discontinuity in the magnetic field close to the boundary between the hot spot and the rest of the unit cell at 3005 fs (smaller rectangle in the center). The magnetic field in the hot spot at 3005 fs retains the spatial pattern of the field at 3000 fs. This is the rectified quasistatic magnetic field produced by the TI (referred to as the QS mode). The fourth panel in Figure 4a shows the magnetic field after approximately 118.5 optical cycles past TI at 5000 fs. We find that, in the absence of losses, the QS mode persists even as the fields from temporally scattered MGW outside the hot spot weaken.

**FIGURE 4:**
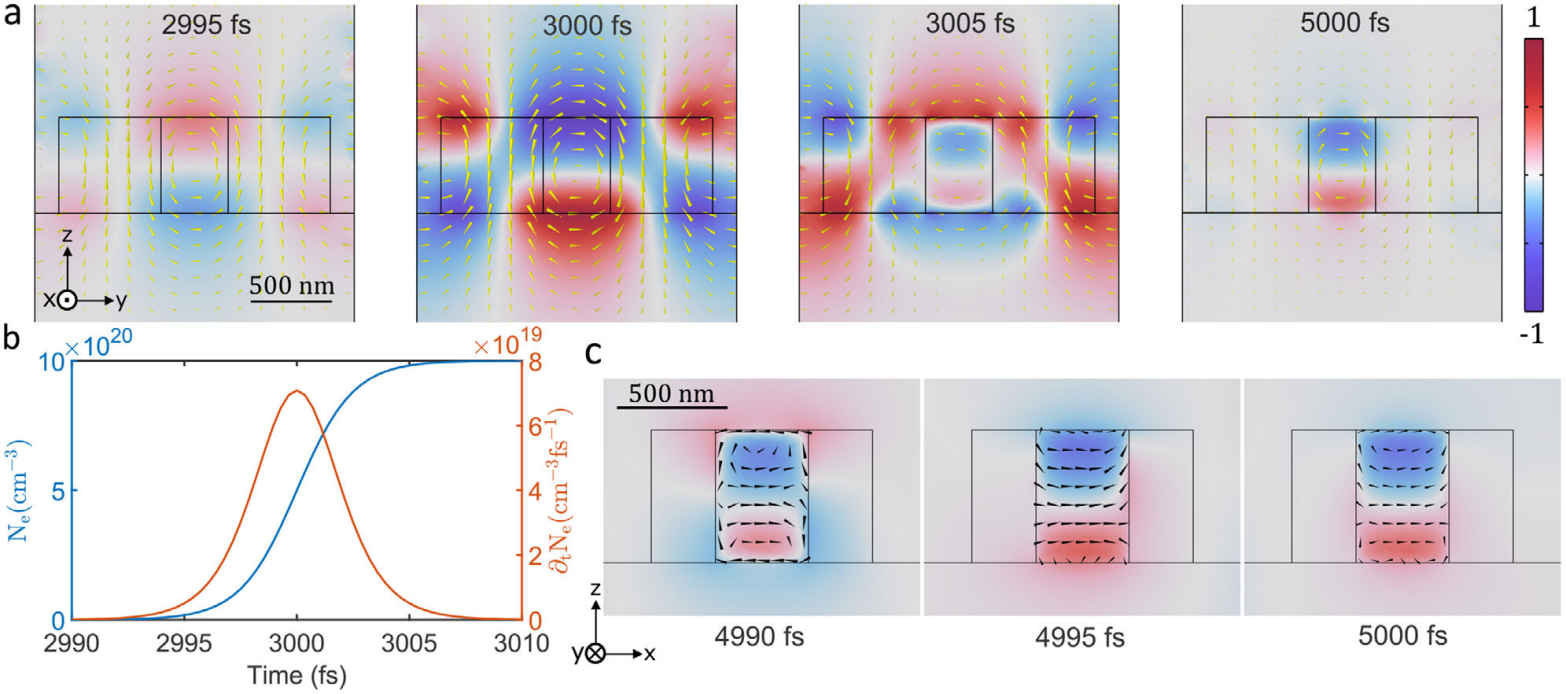
(a) Normalized $H_{y}$ profile in the y‐z mid‐plane of the meta‐atom marked using a light green star in Figure 3b, at 2995, 3000, and 3005 fs; the yellow cones represent the magnetic field lines (b) Time evolution of FC density and its temporal derivative. (c) Time evolution of the QS mode at three consecutive time steps separated by Δt=5fs in the x‐z midplane of the same meta‐atom; the black cones indicate the FC current density in the hot spot. In all plots, the rectangular region within the meta‐atom represents the cylindrical hot spot with radius 210 nm.

Such magnetic rectification can be understood from Equation (6), which shows that the source term for the quasistatic magnetic vector potential $A_{s}$ is a weighted integral of the magnetic vector potential of the MGW during TI where the weights are $\partial_{t}N_{e}^{\left(2\right)}$ (see Figure 4b). The rectified magnetic field in the hot spot at 3005 and at 5000 fs maintains a spatial structure resembling the one at 3000 fs, where $\partial_{t}N_{e}^{\left(2\right)}$ is the highest. The rectification of the magnetic field is not perfectly efficient (∼53% efficiency) due to the finite duration of the time interface (∼16 fs in the case shown in Figure 4). An instantaneous TI at a time when the MGW optical fields are at their peak could enable perfectly efficient rectification, but this is an unphysical scenario [31]. On the other hand, a prolonged TI spanning multiple optical cycles of the MGW would average over multiple cycles, yielding negligible rectification.

Further evidence of the static nature of the QS mode can be seen in Figure 4c from the x‐z midplane snapshots that show the field $H_{y}$ in three time steps separated by $\Delta t=5\;\mathrm{fs}=0.3\;T_{f}$ (where $T_{f}=16.84\;\mathrm{fs}$ is the optical cycle of the frequency‐shifted MGW). Even as the fields outside the hot spot change with time because of the propagation of the temporally scattered MGW at different points in the optical cycle, the QS mode remains unchanged. The electric currents in the hot spot (black cones within the smaller rectangle at the center) replace the displacement currents during the TI and maintain the quasistatic magnetic field, remaining mostly unchanged over the optical cycle of the frequency‐shifted MGW. However, there are small alterations near the boundary of the hot spot where the electrons screen the electric field of the temporally scattered MGW outside the hot spot. The absolute magnitude of the rectified magnetic field in the QS mode, $H_{s}$, depends on the optical fields of the MGW. The maximum optical field of MGW that may propagate along the metasurface is, in turn, limited by the laser‐induced damage threshold of Ge, which has been measured as $1.7\;\mathrm{TW}/{\mathrm{cm}}^{2}$ at 3.6μm [39]. We obtain a peak magnetic field amplitude of ∼2.4T when we excite the MGW such that the resonantly enhanced MGW intensity does not exceed $0.17\;\mathrm{TW}/{\mathrm{cm}}^{2}$ anywhere in the Ge meta‐atom. With ∼53% rectification of the MGW magnetic field for the TI being considered, the peak rectified magnetic field amplitude is ∼1.272T. As can be seen from Equation (6), the magnetic rectification of the resonantly enhanced magnetic field via rapid free‐carrier generation is a robust phenomenon that occurs for all free‐carrier generation ($\partial_{t}N_{e}^{\left(2\right)}>0$). We choose a higher carrier density to clearly demonstrate the rectified magnetic field as the oscillating MGW fields are expelled from the hot spot, making it easier for the rectified currents to circulate and support the quasistatic magnetic fields. Furthermore, a scheme for experimental verification of the quasistatic magnetic field could be developed in which we completely metalize all the meta‐atoms and detect the magneto‐optically induced polarization rotation of a secondary pulse incident on the metasurface immediately after the time interface [60, 61]. The supplementary video shows complete metallization of all the meta‐atoms, which requires a single pump pulse. Upon complete metallization, we observe similar enhancements in the rectified magnetic fields to those observed in localized metallization. Thus, we find that rectification of a resonantly enhanced AC magnetic field by a TI via LFCG offers a new paradigm for realizing giant, nanoscale magnetization without an external magnetic field.

#### 4.3.2 Dissipative Losses and Spatially Inhomogeneous FC Generation

In the absence of losses, the rectified magnetic field persists, and its magnitude remains constant. However, the persistence of the QS mode is hindered by the disruption of quasistatic currents arising from electron, ion, and phonon scattering. We consider an arbitrary electronic‐scattering frequency, $\gamma^{-1}=100$ fs, which encapsulates contributions from various scattering mechanisms. This leads to a modification of Equation (4) where a damping term is introduced as $\partial_{t}^{2}P_{e}+\gamma \partial_{t}P_{e}=\varepsilon_{0}\omega_{p}^{2}\left(t\right)E$. Due to these losses, the electronic currents that support the quasistatic magnetic field eventually decay, and the zero‐magnetic‐field region near the center of the hot spot diffuses until it occupies the entire hot spot. This magnetic diffusion may be modeled using the magnetic diffusion equation, $\partial_{t}H=\left(1/\mu_{0}\sigma_{0}\right)\nabla^{2}H=D\;\nabla^{2}H$ [62], where the electronic diffusion constant $D=1/\mu_{0}\sigma_{0}$ and $\sigma_{0}$ is the electrical DC conductivity, which is inversely proportional to γ.

Thus far, we had assumed that the hot spot was a homogeneous cylinder with radius $r_{\mathrm{hs}}$ with constant $N_{e}$. Now, we account for spatially inhomogeneous FC generation arising from the pump pulse's transverse Gaussian intensity profile by using the Keldysh model (see Section S5). The spatially inhomogeneous FC density profile obtained is shown in Figure 5a. Furthermore, to quantify the persistence of the QS mode after the TI, we focus on the rectified quasistatic magnetic field and consider the difference between its peak positive and peak negative magnetic fields. We consider two regions in the x‐z midplane inside the hot spot where the QS mode is dominant over the MGW as shown in Figure 5b and calculate $\Delta H_{\mathrm{QS}}$ by subtracting the minimum magnetic field amplitude in the region enclosed by the dotted green line from the maximum magnetic field amplitude in the region enclosed by the dotted black line. Then, we define a normalized quantity, $\eta_{\mathrm{QS}}\left(t\right)=\Delta H_{\mathrm{QS}}\left(t\right)/\max.(\Delta H_{\mathrm{QS}}\left(t\right))$.

**FIGURE 5:**
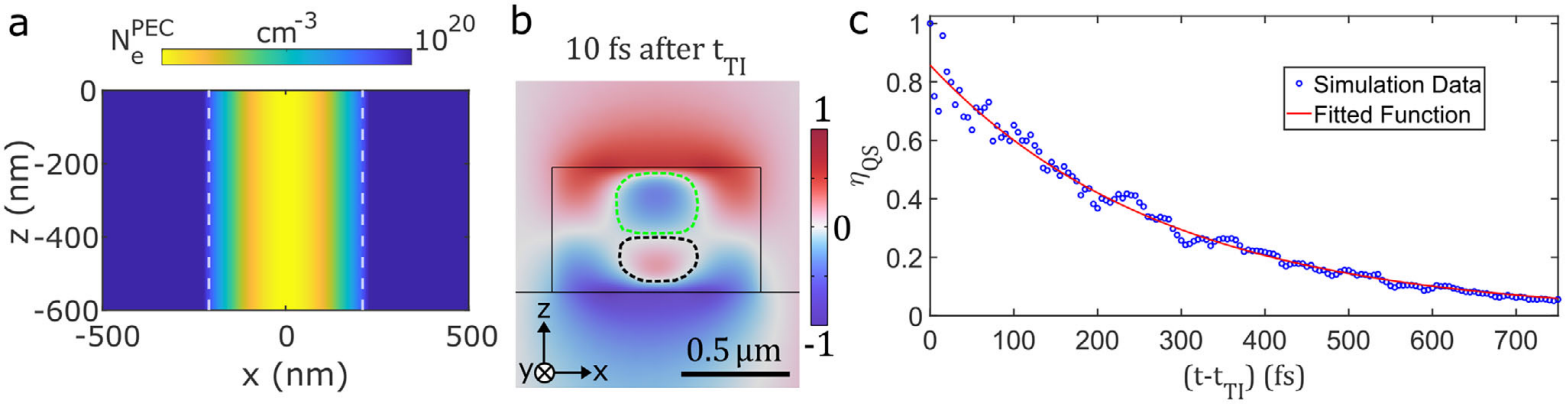
(a) Spatially inhomogeneous distribution of free carriers in the hot spot obtained from the Keldysh model. (b) The rectified magnetic field 10 fs after the TI. The dashed polygons enclose the regions inside the hot spot used to calculate $\Delta H_{\mathrm{QS}}\left(t\right)$. (c) $\eta_{\mathrm{QS}}$ after the time interface, the open circles show the simulation data, while the red line shows the exponential decay function, $\eta_{\mathrm{fit}}$, fitted to the simulation data.

Thus, lower values of $\eta_{\mathrm{QS}}$ correspond to smaller amplitudes of the QS mode at the hot spot. We fit the simulation data for $\eta_{\mathrm{QS}}$ using an exponential decay function that accounts for the diffusion of $H_{s}$ in the presence of losses, $\eta_{\mathrm{fit}}=C_{0}e^{-\gamma_{\mathrm{diff}}t}$. In Figure 5c, which shows the temporal evolution of $\eta_{\mathrm{QS}}$, the open circles represent the simulation data points and the red line represents the fitted function. From the fitted function, we obtain $C_{0}=0.84$, and $\gamma_{\mathrm{diff}}=3.306\times 10^{12}\;\mathrm{rad}/s$ and calculate the diffusion time of the static currents inside the hot spot, $T_{\mathrm{diff}}=\gamma_{\mathrm{diff}}^{-1}=302.48$ fs. Thus, the rectified field persists for more than three electronic scattering times, extending for nearly 20 optical cycles of the incident MGW. However, the persistence of the QS mode is fundamentally limited by the electron‐scattering time, which can be increased by working at lower temperatures as the electron mobility in Ge scales with temperature T as $\mu \propto \gamma^{-1}\propto T^{-1.66}$ [63]. Thus, the 100 fs scattering time we have assumed at room temperature would increase to ∼956 fs at 77 K; consequently, the diffusion time of the QS mode can be extended to ∼3 ps or longer by operating at cryogenic temperatures, thereby improving its persistence.

## 5 Conclusion

In conclusion, we have presented a method for tunable shifting of a high‐Q resonance in an all‐dielectric metasurface resonant in the mid‐IR by controlled LFCG in a cylindrical hot‐spot region within the meta‐atoms. We derived an analytic expression for the frequency shift, taking into account the full vectorial form of the unperturbed electromagnetic fields integrated over the perturbed volume along with the perturbation to the permittivity, that accurately predicts the blueshift of the metasurface resonance due to FC generation in the hot spot in the perturbative regime. We found that, for low FC densities in the hot spot, the resonance blueshifts. However, a further increase in FC density that effectively metalizes the hot spot ($\varepsilon_{\mathrm{hs}}\ll 0$) results in a strongly red‐shifted resonance frequency. Thus, in an experimental setting, carefully tuning the pump intensity to control the local FC density at the hot spot would allow tunable blue‐ or redshifting of the metasurface resonance.

We have further demonstrated that the shift in the metasurface resonance induced by LFCG provides a platform for studying time‐varying photonics in the mid‐IR. We studied the effect of LFCG across an array of hot spots at the center of each meta‐atom, where we calculated the FC generated by a pump pulse using the Keldysh model. Modeling each hot spot as a time‐dependent Drude–Lorentz medium with rapidly varying plasma density, we developed a theoretical framework to incorporate the effects of rapid free‐carrier generation over short but finite time scales into time‐domain electromagnetic codes. When implementing a moderately sharp TI using LFCG for a mid‐IR MGW, we observed a redshift in the temporally scattered MGWs. Furthermore, using Poynting's Theorem, we identified various contributions to the total energy and derived an analytic expression for the total energy density in a time‐varying Drude–Lorentz medium. We demonstrated the re‐partitioning of the incident MGW energy into temporally scattered MGW and persistent electric currents and magnetic fields in the domain where LFCG occurs. By comparing cases with and without a TI, we confirmed that the system's total energy remains unchanged in the presence of a TI and that LFCG does not act as an energy source or sink. Additionally, after TI, a large, highly localized, and persistent quasistatic magnetic field (QS mode) is observed in the hot spot, which does not radiate but consumes a substantial portion of the electromagnetic energy. Using the TI created by LFCG, we provided evidence of efficient rectification (∼53%) of the resonantly enhanced AC magnetic field due to the MGW at the hot spot. This shows that a time interface, realized via rapid FC generation in an all‐dielectric metasurface, can serve as a novel platform for the efficient conversion of an AC optical field at frequency ω into a DC optical field. These results significantly enhance and clarify our understanding of energy dynamics in a time‐varying medium and of magnetic‐field rectification at a TI. We are optimistic that our findings will open new research directions in optically tunable metasurfaces as a platform for time‐varying photonics.

## Conflicts of Interest

The authors declare no conflicts of interest.

## Supporting information


**Supporting File 1**: advs76635‐sup‐0001‐SuppMat.pdf.

**Supplemental File 2**: advs76635‐sup‐0002‐VideoS1.mp4.

## Data Availability

The data that support the findings of this study are available from the corresponding author upon reasonable request.
