# Supplementary material for: Generation and Enhancement of Persistent Nanoscale Magnetization in All‐Dielectric Metasurfaces by Optically Injected and Localized Free Carriers
Source: Adv Sci (Weinh). 2026 Aug 3;13(56):e76635. doi: 10.1002/advs.76635 (PMC13430411; doi:10.1002/advs.76635)
Supplement: Supplementary file 1 [file ADVS-13-e76635-s001.pdf]

Supplementary Information: Generation and Enhancement of Persistent Nanoscale Magnetization in All-Dielectric Metasurfaces by Optically Injected and Localized Free Carriers

Shivaksh Rawat[*,1], Samyobrata Mukherjee[1], and Gennady Shvets[*,1]

[1]School of Applied and Engineering Physics, Cornell University, Ithaca, NY 14850, USA
[*]Email: sr939@cornell.edu, gshvets@cornell.edu

S1 Predicting Resonance Frequency Shifts

The real unperturbed ($\mathbf{F}_0$) and perturbed ($\mathbf{F}'$) fields inside the resonator are

$$\mathrm{Re}(\mathbf{F}_0) = \mathbf{F}_0(\mathbf{r})\mathrm{e}^{-i\omega_0 \mathrm{t}}\text{+c.c.}; \quad \mathrm{Re}(\mathbf{F}') = \mathbf{F}'(\mathbf{r})\mathrm{e}^{-i\omega \mathrm{t}}\text{+c.c.} \tag{1}$$

where $\mathbf{F}$ represents the complex fields $\mathbf{E}, \mathbf{D}, \mathbf{H}$, and $\mathbf{B}$. Here, $\mathbf{F}_0(\mathbf{r})$ and $\mathbf{F}'(\mathbf{r})$ are complex field amplitudes, which are functions of position. Subtracting the scalar product of Maxwell's curl equation for $\mathbf{H}'$ with $\mathbf{E}_0^*$ from the scalar product of the curl equation for $\mathbf{E}'$ with $\mathbf{H}_0^*$ yields the following results.

$$\mathbf{H}_0^* \cdot (\nabla \times \mathbf{E}') - \mathbf{E}_0^* \cdot (\nabla \times \mathbf{H}') = i\omega \mathbf{B}' \cdot \mathbf{H}_0^* + i\omega \mathbf{D}' \cdot \mathbf{E}_0^*, \tag{2}$$

where ω is the perturbed complex resonance frequency, and "$*$" represents the complex conjugate. The LHS of eq. (2) can be rewritten using the divergence formula as

$$\mathbf{H}_0^* \cdot (\nabla \times \mathbf{E}') - \mathbf{E}_0^* \cdot (\nabla \times \mathbf{H}') = \nabla \cdot (\mathbf{E}_0^* \times \mathbf{H}' - \mathbf{H}_0^* \times \mathbf{E}') + \mathbf{E}' \cdot (\nabla \times \mathbf{H}_0^*) - \mathbf{H}' \cdot (\nabla \times \mathbf{E}_0^*) \tag{3}$$

Using the identity in eq. (3) and $\omega = \omega_0 + \Delta\omega$ (where ω_0 is the unperturbed complex resonance frequency and $\Delta\omega$ is the change in resonance frequency due to perturbation) to simplify eq. (2) yields the following.

$$i\Delta\omega(\mathbf{D}' \cdot \mathbf{E}_0^* + \mathbf{B}' \cdot \mathbf{H}_0^*) = i\omega_0[(\mathbf{D}_0^* \cdot \mathbf{E}' - \mathbf{D}' \cdot \mathbf{E}_0^*) - (\mathbf{B}' \cdot \mathbf{H}_0^* - \mathbf{B}_0^* \cdot \mathbf{H}')] + \nabla \cdot (\mathbf{E}_0^* \times \mathbf{H}' - \mathbf{H}_0^* \times \mathbf{E}') \tag{4}$$

Integrating over the entire unit cell, we obtain the following.

$$\begin{aligned}\int_{\mathrm{V}} \Delta\omega(\mathbf{D}' \cdot \mathbf{E}_0^* + \mathbf{B}' \cdot \mathbf{H}_0^*)\mathrm{dV} = \int_{\mathrm{V}} \omega_0[(\mathbf{D}_0^* \cdot \mathbf{E}' - \mathbf{D}' \cdot \mathbf{E}_0^*) - (\mathbf{B}' \cdot \mathbf{H}_0^* - \mathbf{B}_0^* \cdot \mathbf{H}')]\mathrm{dV} \\ -i\int_{\mathrm{V}} \nabla \cdot (\mathbf{E}_0^* \times \mathbf{H}' - \mathbf{H}_0^* \times \mathbf{E}')\mathrm{dV}\end{aligned} \tag{5}$$

Using Gauss's theorem, the integral with the divergence term (second term on the RHS) in eq. 5 can be rewritten as a surface integral evaluated on the surface S of the unit cells of the metasurface,

$$-i\int_{\mathrm{V}} \nabla \cdot [\mathbf{E}_0^* \times \mathbf{H}' - \mathbf{H}_0^* \times \mathbf{E}']\mathrm{dV} = -i\oint_{\mathrm{S}} \hat{\mathrm{n}} \cdot [\mathbf{E}_0^* \times \mathbf{H}' + \mathbf{E}' \times \mathbf{H}_0^*]\mathrm{dS} \tag{6}$$

where $\hat{\mathrm{n}}$ is the unit normal to the surface S. Furthermore, we assume that the perturbation in the hot spot at the center of the meta-atom has negligible impact on the fields at the unit cell walls, so that $\mathbf{E}'|_{\mathrm{walls}} \approx \mathbf{E}_0|_{\mathrm{walls}}$ and $\mathbf{H}'|_{\mathrm{walls}} \approx \mathbf{H}_0|_{\mathrm{walls}}$. Under this assumption, the RHS of eq. 6 may be written as

$$-i\oint_{\mathrm{S}} \hat{\mathrm{n}} \cdot [\mathbf{E}_0^* \times \mathbf{H}' + \mathbf{E}' \times \mathbf{H}_0^*]\mathrm{dS} \approx -2i\oint_{\mathrm{S}} \hat{\mathrm{n}} \cdot \mathrm{Re}(\mathbf{E}_0 \times \mathbf{H}_0^*)\mathrm{dS} = -4i\oint_{\mathrm{S}} (\hat{\mathrm{n}} \cdot \mathbf{S}_0)\mathrm{dS} \tag{7}$$

where $\mathbf{S}_0$ is the Poynting vector and thus is purely real. Therefore, the surface integral in eq.(7) calculates the change in the imaginary part of the resonance frequency ($\mathrm{Im}(\Delta\omega)$), which is also related to the quality

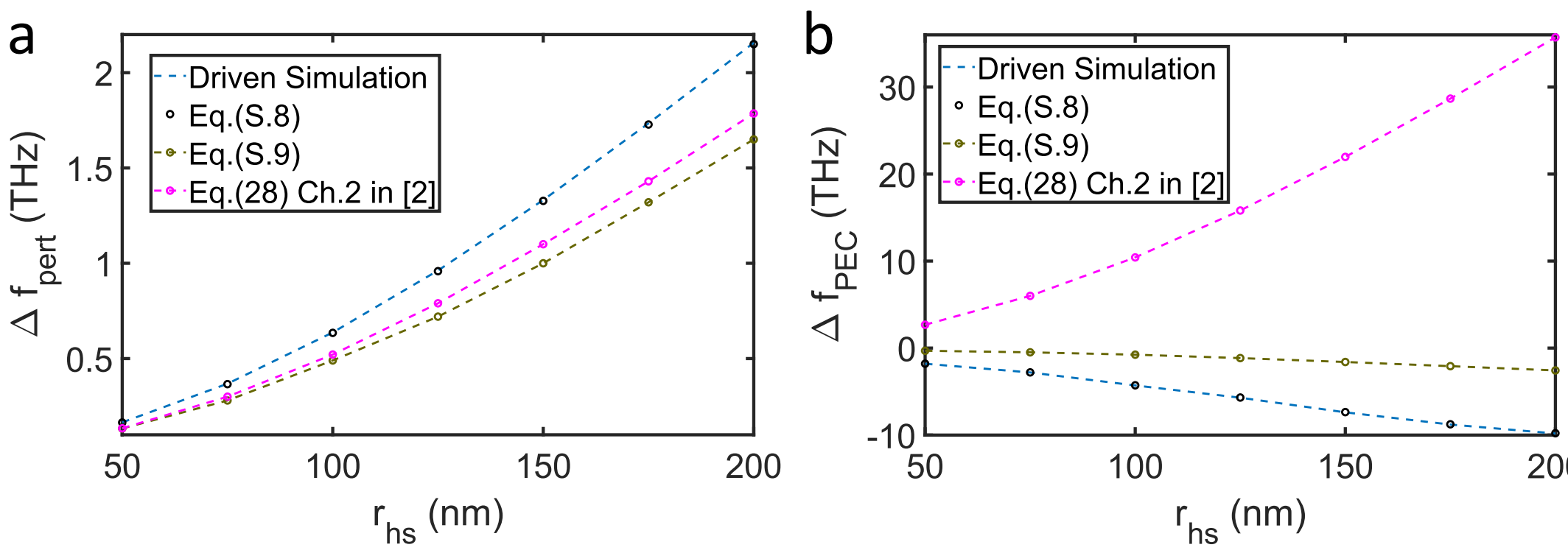

Figure S1: Comparison of the resonance frequency shift predictions for **a**. $\mathrm{N_e} = \mathrm{N_e^{pert}} = 1.6 \times 10^{19}\mathrm{cm^{-3}}$, and **b**. $\mathrm{N_e} = \mathrm{N_e^{PEC}} = 3.2 \times 10^{20}\mathrm{cm^{-3}}$ for increasing hot spot radii.

factor of the metasurface resonance. However, since we are only interested in calculating the change in the real part of the resonance frequency ($\mathrm{Re}(\Delta\omega)$), we neglect the surface-integral term to obtain

$$\frac{\mathrm{Re}(\Delta\omega)}{\mathrm{Re}(\omega_0)} = \frac{\int_{\mathrm{V}}[(\mathbf{D}_0^* \cdot \mathbf{E}' - \mathbf{D}' \cdot \mathbf{E}_0^*) - (\mathbf{B}' \cdot \mathbf{H}_0^* - \mathbf{B}_0^* \cdot \mathbf{H}')]\mathrm{dV}}{\int_{\mathrm{V}}(\mathbf{D}' \cdot \mathbf{E}_0^* + \mathbf{B}' \cdot \mathbf{H}_0^*)\ \mathrm{dV}} \tag{8}$$

where ω_0 is the resonance frequency of the unperturbed metasurface (which is complex for typically leaky metasurface resonances with the imaginary part related to the decay of the resonant fields), and V is the volume of the metasurface unit cell. Eq.(8) accurately predicts the shifts in the resonance frequency for any perturbation to the meta-atom. However, it is of limited use since it requires knowledge of the complete fields in the perturbed structure, which are generally unknown. To simplify Eq.(8) to the lowest order in the perturbed fields, we assume that the fields in the perturbed volume (hot spot) is represented by a small cylinder of radius r_{hs}, as shown in Fig. 1c of the main text, which can be broken up into infinitesimally thin annular shells with radii $0 < \rho < \mathrm{r_{hs}}$, thicknesses $\mathrm{d}\rho$, and area S_ρ. Equation (8) may be further simplified by decomposing the fields into normal ($\perp$) and tangential ($\|$) components with respect to the surface $'\mathrm{S}'$ of the infinitesimally thin annular cylinder. Since $\mathbf{D}_\perp$, $\mathbf{E}_\|$, $\mathbf{H}_\perp$, and $\mathbf{B}_\|$ are continuous across an interface, all the fields in eq. (8) are expressed in terms of these continuous components: $\mathbf{D}_0 = \epsilon_0\varepsilon_{\mathrm{ini}}\mathbf{E}_{0\|} + \mathbf{D}_{0\perp}$, and $\mathbf{E}' = \mathbf{E}'_\| + (1/\epsilon_0\varepsilon_{\mathrm{fin}})\mathbf{D}'_\perp$. The second term inside the integral in the numerator of 8 vanishes because the magnetic permeability is unchanged. Furthermore, since we consider small changes in permittivity and infinitesimally thin shells, we assume that $\mathbf{E}'_\|(\rho) = \mathbf{E}_{0\|}(\rho)$ and $\mathbf{D}'_\perp(\rho) = \mathbf{D}_{0\perp}(\rho)$ (azimuthal angle ϕ dependence and the variation of fields in the z direction have been implicitly assumed). Keeping only the unperturbed fields in the denominator of Eq. 8, the following simplified expression for the frequency shift is obtained:

$$\frac{\mathrm{Re}(\Delta \mathrm{f})}{\mathrm{Re}(\mathrm{f_0})} = \frac{\int_0^{\mathrm{r_{hs}}} \left\{ \oint_{\mathrm{S}} \epsilon_0 \left([\varepsilon_{\mathrm{ini}} - \varepsilon_{\mathrm{fin}}] |\mathbf{E}_{0\|}(\rho)|^2 - \left[\frac{1}{\varepsilon_{\mathrm{ini}}} - \frac{1}{\varepsilon_{\mathrm{fin}}} \right] |\mathbf{D}_{0\perp}(\rho)|^2 \right) d\mathrm{S} \right\} \mathrm{d}\rho}{\int_{\mathrm{V}}(\mathbf{D}_0 \cdot \mathbf{E}_0^* + \mathbf{B}_0 \cdot \mathbf{H}_0^*)\ \mathrm{dV}} \tag{9}$$

where f is the frequency in THz. The resonance frequency shifts predicted by eq. (8), and eq. (9) are then compared to driven simulations in COMSOL Multiphysics and also with the resonance frequency shifts predicted using eq. (28) in chapter 2 of ref. [1] for reference. Fig. S1a, and S1b show the calculated shift in the metasurface resonance frequency using these various formulae for different hot spot radii ($\mathrm{r_{hs}}$) when $\mathrm{N_e} = \mathrm{N_e^{pert}} = 1.6 \times 10^{19}\mathrm{cm^{-3}}$, and $\mathrm{N_e} = \mathrm{N_e^{PEC}} = 3.2 \times 10^{20}\mathrm{cm^{-3}}$, respectively. The shifts predicted by eq. (8), using knowledge of the fields in the perturbed system, are the most accurate and in accordance with the COMSOL simulations. From Fig. S1a, we observe that the resonance frequency shifts predicted from eq.(9) using only the unperturbed fields are reasonably accurate and are similar to the shifts predicted using eq. (28) in chapter 2 of Ref. [1] for the case of small perturbation (i.e., Fig. S1a, where $\mathrm{N_e} = \mathrm{N_e^{pert}}$). For large perturbations, when the hot spot permittivity becomes negative (i.e., Fig. S1b, where $\mathrm{N_e} = \mathrm{N_e^{PEC}}$), eq. (8) is still the most accurate; however, in this case, eq. (9) predicts shifts on the correct side of the unperturbed frequency while eq. (28) in chapter 2 of Ref. [1] does not. This shows that eq. (9) can accurately predict the nature of the resonance frequency shifts for all perturbations.

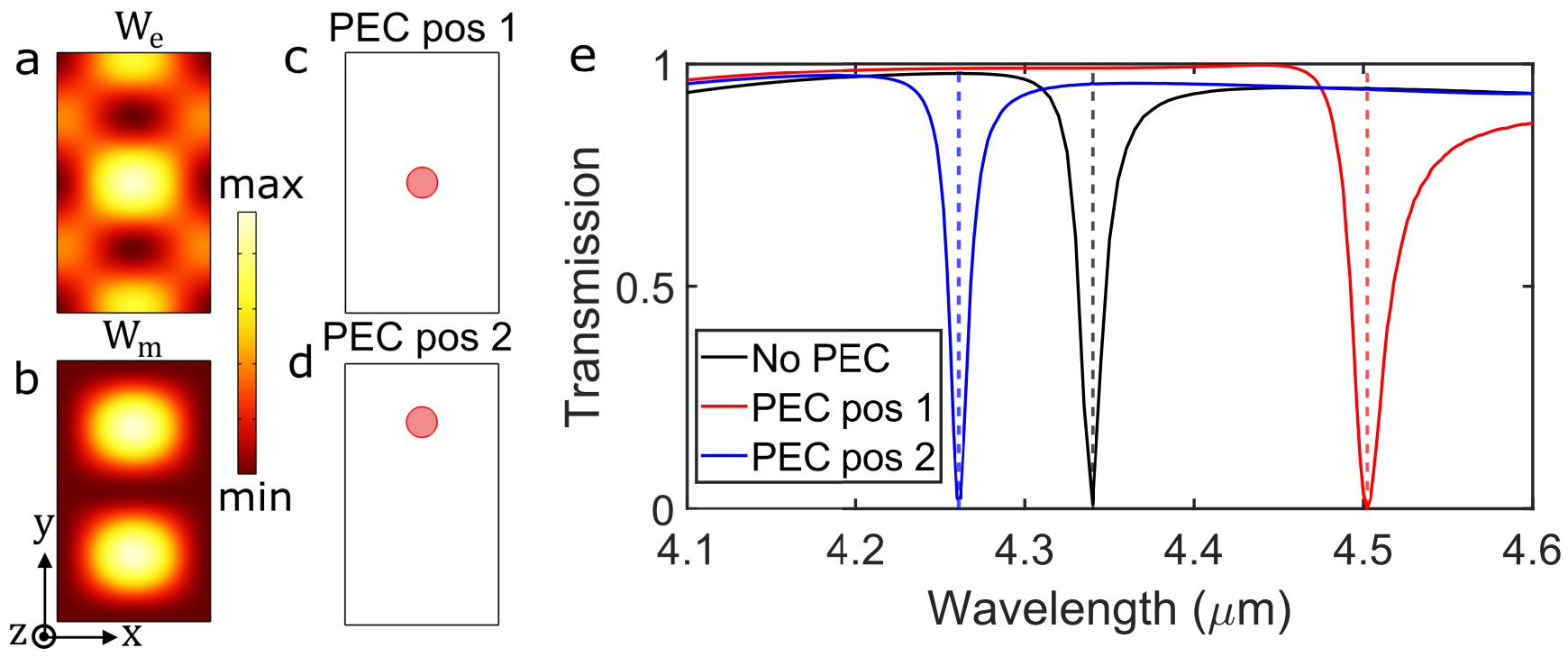

Figure S2: **a**. (**b**.) Normalized electric (magnetic) energy density of the ED resonance for the unperturbed metasurface. **c**. Schematic showing an effectively metalized (PEC) but smaller hot spot (r_{hs} = 100nm) placed in the center of the Ge block, where W_e is dominant (position 1). **d**. Same as **c** but with the hot spot now placed in a location, where W_m is dominant (position 2). **e**. Metasurface transmission spectrum for the cases where the metasurface is unperturbed (black), PEC is in position 1 (red), and PEC is in position 2 (blue).

S2 Manipulating Magnetic Energy Density

The electromagnetic energy density of a resonant mode is unevenly distributed within the meta-atom, with the electric and magnetic energy densities dominant in different spatial regions. Therefore, despite considering a metasurface comprising nonmagnetic material ($\mu_r = 1$), it is possible to modify the magnetic energy density of the resonant mode, e.g., by effectively metalizing or introducing PEC in a specific region where the magnetic energy density dominates the electric energy density. The resulting frequency shift $\Delta\omega_{\mathrm{PEC}}$ of the metasurface resonance is given by

$$\begin{gathered}\frac{\mathrm{Re}(\Delta\omega_{\mathrm{PEC}}^2)}{\mathrm{Re}(\omega_0^2)} = \int_{\Delta \mathrm{V}} \left(\mu_0 \left|\mathbf{H}_0\right|^2 - \epsilon_0\varepsilon_\infty \left|\mathbf{E}_0\right|^2\right) \mathrm{dV} \\ \int_{\mathrm{V}} \mathbf{H}_0^*.\mathbf{H}_0 \mathrm{dV} = \frac{1}{\mu_0}, \quad \int_{\mathrm{V}} \mathbf{E}_0^*.\mathbf{E}_0 \mathrm{dV} = \frac{1}{\epsilon_0\varepsilon_\infty}\end{gathered} \tag{10}$$

where $\mathbf{E}_0$ and $\mathbf{H}_0$ are the normalized unperturbed electric and magnetic fields, respectively, and ΔV is the PEC volume inside each meta-atom. Assuming a dispersion-free high-frequency relative permittivity for Ge (i.e., $\mathrm{d}\varepsilon_\infty/\mathrm{d}\omega = 0$) and absence of free carriers ($\omega_p = 0$), the above choice of normalization gives the magnetic and electric energy density as $W_m = \mu_0|\mathbf{H}_0|^2/2$ and $W_e = \epsilon_0\varepsilon_\infty|\mathbf{E}_0|^2/2$, respectively. According to Eq. (10) in the main text, the introduction of a PEC in a region of a resonator where the magnetic (electric) energy density is dominant would result in a blue shift (red shift) of the resonance [2, 3].

Thus, when a strongly metallized (PEC) but smaller hot spot of radius r_{hs} =100 nm is introduced in the center of the Ge block (Fig. S2c), it occupies a region of high electric energy density. This expels out the electric field from the PEC volume and increases the effective capacitance while redshifting the ED resonance from 4.34 μm to 4.5 μm as shown in Fig. S2e. We have seen this effect in the previous sections, but note that our choice of a smaller hot spot region reduces the extent of the redshift of the ED resonance. However, when a PEC is introduced in an off-center region of the Ge block where the magnetic energy density is dominant (Fig. S2d), the resonance blueshifts to 4.26 μm (Fig. S2e). Since the PEC now expels the magnetic field, it reduces the effective inductance of the meta-atom due to excessive FCs that shield the magnetic flux, resulting in a blueshift of the ED resonance.

S3 Meta-atom Homogenization: Effective Permittivity Model

In this section, we provide further insight into the behavior of the metasurface resonance upon FC generation, particularly the redshifting of the ED resonance at high values of $N_e > 10^{20}$ cm^{-3}. We employ an electrostatic capacitor model [4, 5] to homogenize the Ge resonator and the hot spot and calculate its effective permittivity ($\varepsilon_{\mathrm{eff}}$). The Ge block and the hot spot are modeled as a parallel plate capacitor, and we calculate the total charge (Q) accumulated on the two longer faces ($\parallel$ to the y-z

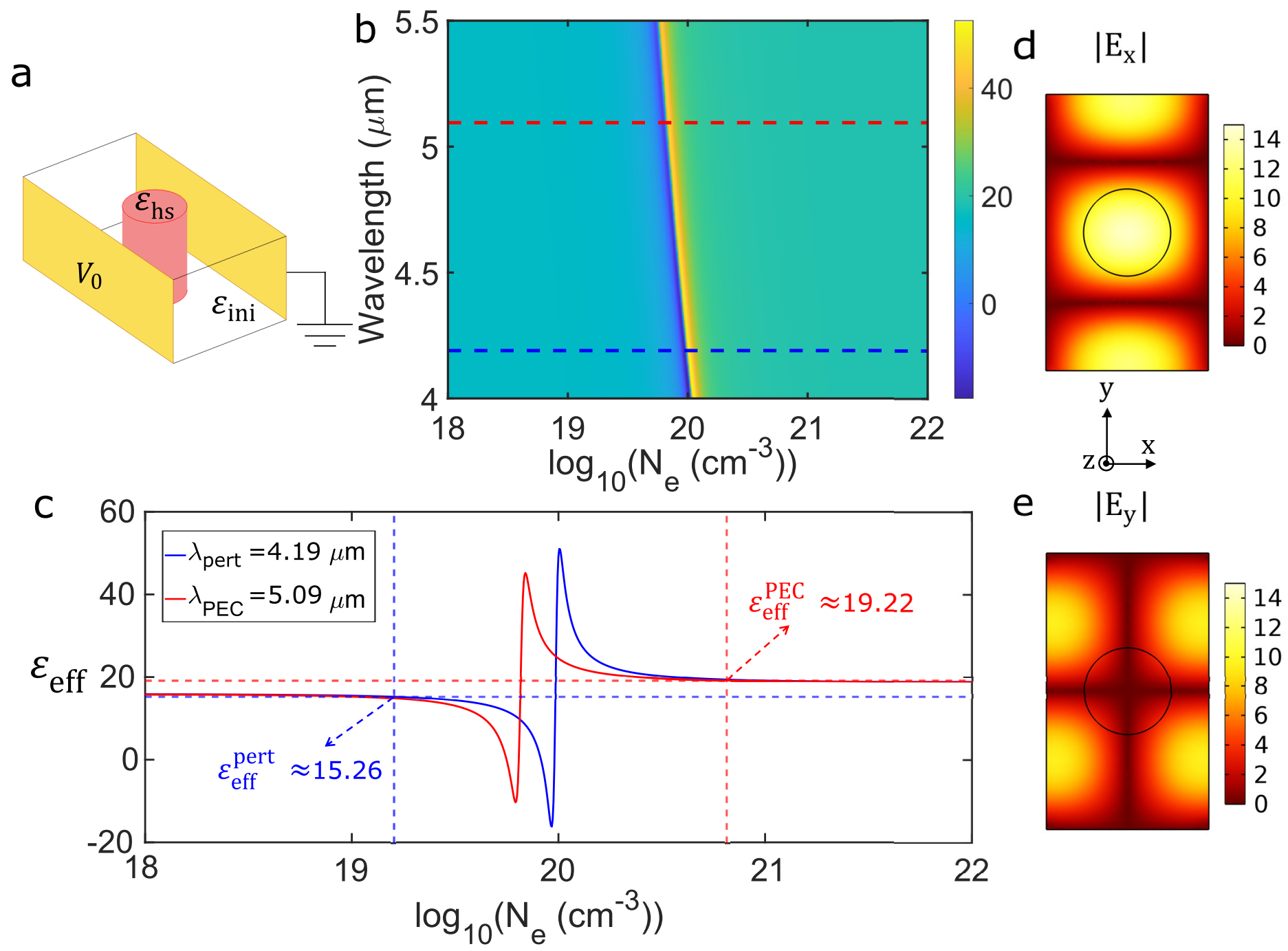

Figure S3: **a**. Geometry for the effective capacitor model. The parallel plates are shown as golden surfaces, and the hot spot at the center is red. **b**. The effective permittivity of the Ge block as a function of local hot spot carrier density and wavelength. The black, blue, and red dashed lines in **c**. represent the λ_i, λ_{pert}, and λ_{PEC} wavelengths, respectively. **c**. ε_{eff}^{xx} as a function of N_e for two different values of $\lambda = \lambda_{pert}$ (blue) and $\lambda = \lambda_{PEC}$ (red). Note that $\varepsilon_{eff}^{pert} < \varepsilon_{ini} \approx 16 < \varepsilon_{eff}^{PEC}$. **d**. $|E_x|$ field enhancement in the Ge block with the hot spot region marked using a bold circle. **e**. Same as **d** but for $|E_y|$.

plane) when a constant potential difference V_0 is applied across them. The capacitor plates have an area $A = w_y \times h$ with separation w_x as shown in Fig. S3a. The longer sides of the meta-atom are chosen as the parallel plates of the capacitor in our model because changes in the permittivity of the hot spot located in the center of the Ge block affect E_x more than E_y. This can be ascribed to the fact that the maximum (minimum) of $|E_x|$ ($|E_y|$) coincides with the location of the hot spot, as shown in Fig. S3d(e). Then, after calculating Q, we obtain ε_{eff}^{xx} for the x-polarized incidence

$$\varepsilon_{eff}^{xx} = \frac{Qw_x}{\epsilon_0 w_y h V_0}. \tag{11}$$

Fig. S3b shows the value of ε_{eff}^{xx} calculated using the capacitor model for the range of N_e and λ studied in Fig. 2a of the main text. Fig. S3c shows the line cuts of Fig. S3b where we plot the variation in ε_{eff} as a function of N_e for three different wavelengths λ_i (black), λ_{pert} (blue) and λ_{PEC} (red). For all the values of λ, we find that at low values of N_e, $\varepsilon_{eff}^{xx} = \varepsilon_{eff}^{i} = \varepsilon_\infty$. ε_{eff}^{xx} rapidly decreases from ε_∞ to 0 for 10^{19} cm^{-3} $\leq N_e \leq 10^{20}$ cm^{-3}. For values of $N_e \geq 10^{20}$ cm^{-3}, while the local permittivity of the hot spot becomes negative, the effective permittivity of the homogenized medium ε_{eff}^{xx} increases rapidly and saturates at approximately $\varepsilon_{eff}^{xx} \approx 19$. The changes in ε_{eff}^{xx} as N_e crosses $\sim 10^{20}$ cm^{-3} account for changes in the metasurface resonance wavelength seen in Fig. 2a of the main text as N_e crosses 10^{20} cm^{-3} since the resonance wavelength $\lambda_r \propto \sqrt{C_{eff}} \propto \sqrt{\varepsilon_{eff}^{xx}}$ where C_{eff} is the effective capacitance. The intersection of the dashed blue (red) lines shows the value of the effective permittivity for $\lambda = \lambda_{pert}$ (λ_{PEC}) and $N_e = N_e^{pert}$ (N_e^{PEC}). Thus, localized FC generation in the hot spots initially decreases the effective permittivity of the meta-atom and blue-shifts the metasurface resonance. Further increasing the FC density creates a localized region of high negative permittivity (ε_{hs}), thereby increasing the effective permittivity and red-shifting the metasurface resonance. Since the effective capacitor model is electrostatic, it can only be used to determine the effective permittivity for modifications that primarily affect the electric energy.

S4 Keldysh Photoionization Model

We used the Keldysh model [6] to determine the time evolution of the FC density ($N_e(\mathbf{r}, t)$) inside the hot spot as a consequence of illumination by a high-intensity, ultra-short pump pulse. We modeled the laser-induced ionization of Ge by calculating the FC density due to the photoionization process (R_{PI}),

$$\frac{\partial N_e(\mathbf{r}, t)}{\partial t} = R_{PI}(I[t]) - \frac{N_e(\mathbf{r}, t)}{\tau_{e-h}} \tag{12}$$

where I[t] is the time-dependent intensity of the pump pulse and τ_{e-h} is the electron-hole recombination time. Here, we consider photoionization as the primary mechanism for free-carrier generation in Ge; other processes, such as impact/avalanche ionization by the photoionized electrons, will be included in our future calculations. The pump pulse parameters and the germanium optical properties are shown in tabular form below [7, 8, 9].

**Parameter**	**Symbol**	**Value**
Pump wavelength	λ_p	1580 nm
Pulse width	τ_p	8 fs
Band Gap of Ge	Δ	0.8 eV
Reduced effective mass	m_e	$0.041\ m_0, \ \ m_0 = 9.11 \times 10^{-31}$ kg
Refractive index (at λ)	n_0	4.2
Pump Irradiance	I[t]	$I[t] = 2I_{avg}e^{-(t-\tau_2)^2/\tau_p^2}, \ \tau_2 = 3$ ps
e-h Recombination time	τ_{e-h}	100 ps

The photoionization rate calculated using the Keldysh model is given by [10]

$$R_{PI} = 2.\frac{2\omega}{9\pi}\left(\frac{\sqrt{1+\gamma^2}}{\gamma}.\frac{m_e\omega}{\hbar}\right).Q_k\left(\gamma, \frac{\Delta_{NP}}{\hbar\omega}\right).\exp\left[-\pi\left\langle\frac{\Delta_{NP}}{\hbar\omega}+1\right\rangle.\frac{K(\phi)-E(\phi)}{E(\theta)}\right] \tag{13}$$

where a factor of 2 is introduced to account for the electron spin degeneracy and Q_k represents a slow-varying amplitude function and is written as

$$Q_k(\gamma, x) = \sqrt{\frac{\pi}{2.K(\theta)}}.\sum_{n=0}^{+\infty}\exp\left[-\pi.\frac{K(\phi)-E(\phi)}{E(\theta)}.n\right].\Phi\left[\sqrt{\frac{\pi^2(<x+1>-x+n)}{2.K(\theta).E(\theta)}}\right], \tag{14}$$

where, K(x) and E(x) are complete elliptic integrals. θ, ϕ, and γ are given as

$$\theta = \frac{1}{1+\gamma^2}, \qquad \phi = \frac{\gamma}{1+\gamma^2}, \qquad \gamma = \frac{\omega\sqrt{m\Delta}}{eF} \tag{15}$$

$\Phi(x)$ and Δ_{NP} represent the Dawson integral and the laser-modified band gap, which are given as

$$\Phi(x) = \int_0^x (\chi^2 - x^2)\, d\chi; \quad \Delta_{NP} = \frac{2}{\pi}\Delta.\left[\frac{\sqrt{1+\gamma^2}}{\gamma}.E\left(\frac{1}{\sqrt{1+\gamma^2}}\right)\right], \tag{16}$$

where $\omega = 2\pi c/\lambda_p$ is the angular frequency of the pump pulse, e is the electronic charge, F is the local electric field amplitude, and $< x >$ the integer part of x. The photoionization rate in bulk Ge, calculated using the Keldysh model for different pump irradiances, is shown in Fig. S4. Using a source-driven simulation, we observed that the Ge blocks do not exhibit collective behavior at the pump frequency. Therefore, the photoionization rate for the Ge blocks is assumed to be the same as the rates for bulk germanium. The free-electron concentration in the hot spot for various pump intensities is also shown in Fig. S4.

S5 Simulation Setup and FC Generation

The simulation setup used to implement a TI during MGW propagation is shown in Fig. S5a, where the red volume within each meta-atom denotes the hot spot region. The probe points at which we inspect the signal are marked with yellow circles in Fig. S5a. The red arrows indicate a spatial array of phased dipoles with spacing $d_x = \pi/2k_x$ located at a height $d_y = \lambda_0/4$ above the metasurface with

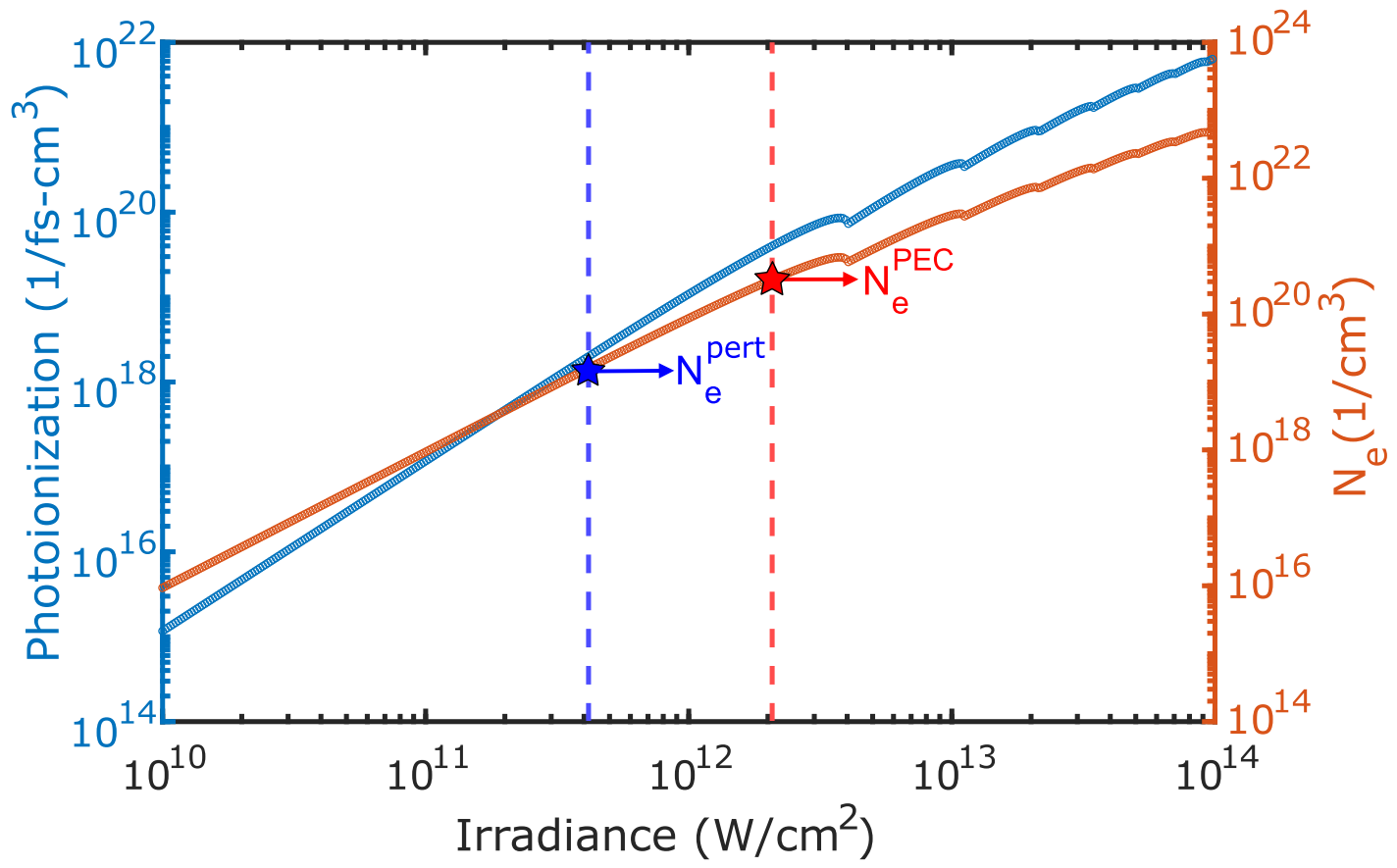

Figure S4: Photoionization (blue) rate for Ge, calculated using the Keldysh model for the given pump pulse parameters. Carrier concentration (red, $\mathrm{N_e}$) as a function of input irradiance; the red (blue) horizontal dashed line represents the average irradiance 2.075 TW/cm^2 (416 GW/cm^2) required for the red-shifting (blue-shifting) of resonance wavelength to λ_{PEC} (λ_{pert}).

dipole moments pointing in the $\hat{z}$ direction. The normalized pump/probe intensities and the temporal evolution of $\mathrm{N_e}$ in the hot spot are shown in the inset of Fig. S5a.

Furthermore, we estimate the spatial inhomogeneity of the FC generation process in the hot spot and use it to accurately model the TI. For the pump pulse parameters used, the Keldysh parameter is less than 1 ($\gamma \ll 1$), indicating that we are in the strong tunneling ionization regime (a highly nonlinear process). Therefore, even when the radius of the focused laser spot on each meta-atom is taken to be diffraction-limited ($\sim \lambda_{\mathrm{p}}/2$), the spatial distribution of the free carriers created does not follow the spatial distribution of the pump intensity in the meta-atom. This implies that the FCs are more strongly localized in the meta-atom due to the nonlinear dependence of the tunneling ionization rate on the pump intensity, which is higher at the center of the focused spot. Additionally, since tunneling ionization is the dominant mechanism for free carrier generation, the hot spot free-carrier density, $\mathrm{N_e}$, is only a function of the pump intensity.

To obtain the spatial distribution of the FCs, we use the Keldysh model to calculate the photoionized FCs created by the pump pulse, whose intensity varies spatially within the meta-atom. On the surface of each meta-atom (i.e., in the x-y plane), we assume a Gaussian distribution of the electric field due to the pump pulse with the beam waist equivalent to $w_0 \sim \lambda_{\mathrm{p}}/4 = 395$ nm (diffraction-limited focused spot)

$$\mathrm{E}(x,y,z) \propto \exp\left(-(x^2+y^2)/w_0^2\right) \Rightarrow \mathrm{I}(x,y,z) = \mathrm{I}_{\mathrm{avg}}^{\mathrm{PEC}} \exp\left(-2(x^2+y^2)/w_0^2\right). \tag{17}$$

where $\mathrm{I}_{\mathrm{avg}}^{\mathrm{PEC}}$ is the average pump intensity required to create a FC density of $\mathrm{N_e^{PEC}}$. We used the spatially varying pump intensity from eq. (17) in the Keldysh model to calculate the spatial dependence of the photoionized electrons and fit them to a Gaussian distribution (see Fig. S5b)

$$\mathrm{N_e}(x,y,z) = \mathrm{N_e^{PEC}} \exp\left(-(x^2+y^2)/\sigma^2\right) \tag{18}$$

Fitting the FC distribution obtained from the Keldysh model to eq. (18), the Gaussian waist of the distribution, $\sigma \sim 210$ nm $\ll w_0$, is taken as the effective radius of the hot spot in all the frequency and time domain simulations (see Fig. S5c). However, we used the complete spatial FC density profile while calculating the effects of losses and spatially inhomogeneous FC generation on the rectified magnetic field in Section 4.3.2 of the main text.

S6 Energy Relations in a Time-Varying Drude-Lorentz Medium

In a time-varying Drude-Lorentz medium, the total electron density $\mathbf{J}_{\mathrm{e}}$ can be written as the sum of pre-existing free electron density $\mathbf{J}_{\mathrm{e}}^{(1)}$ and newly created free electron density $\mathbf{J}_{\mathrm{e}}^{(2)}$ (see Section 3.2 in the

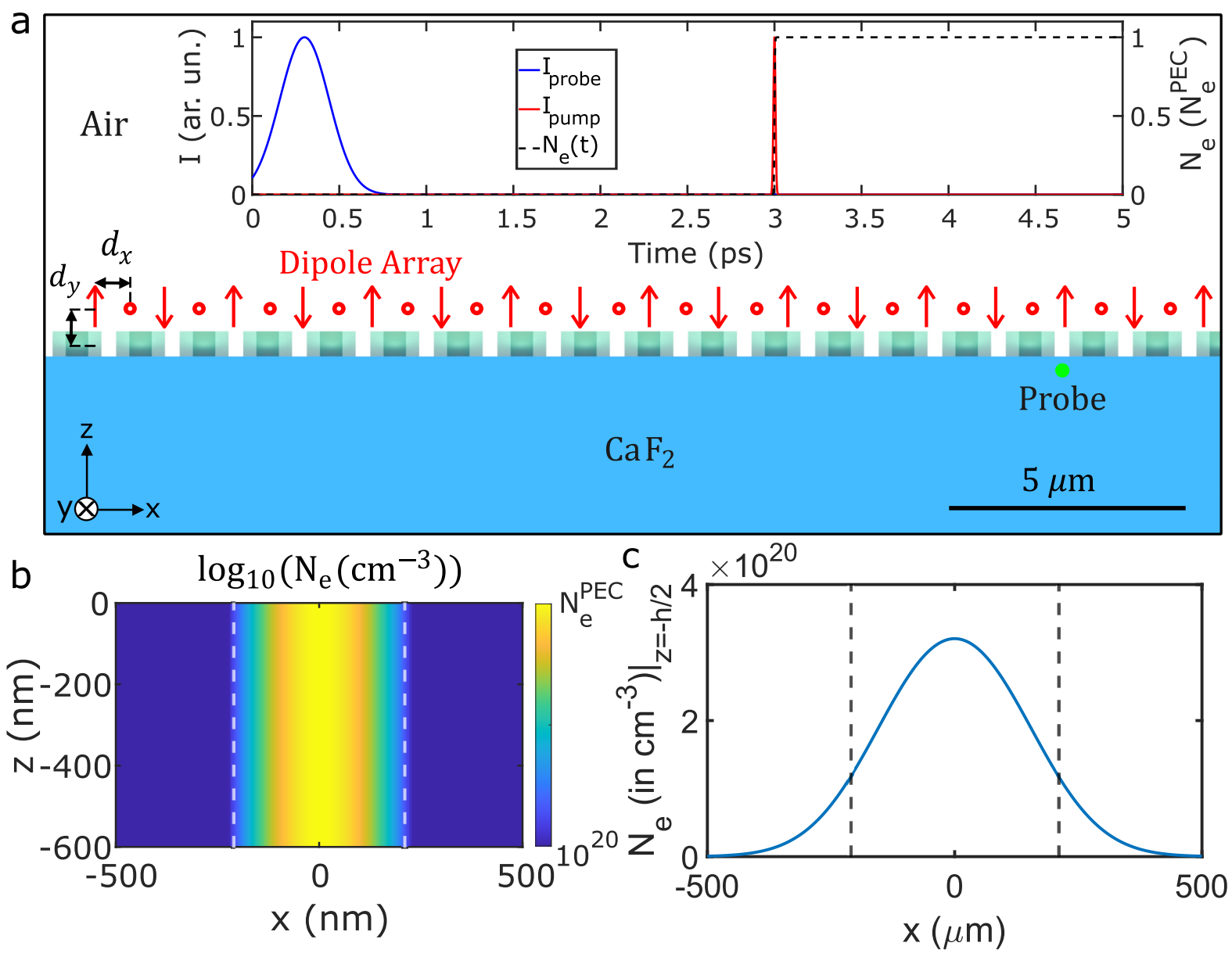

Figure S5: **a**. Setup used for TI simulations where the shaded red region shows the effective cylindrical hot spots of radius 210 nm inside each meta-atom. Probe points are located in the substrate (green circles). MGW is launched using a dipole array (red arrows) separated by $d_x = \pi/2\mathrm{k_x}$ and at a height $d_y = \lambda_0/4$ above the metasurface; the length of the arrows represents the magnitude of the dipole moment. Inset: Pump pulse (red) and dipolar excitation (blue) intensity and the temporal variation of hot spot FC density (black dashed). **b**. Spatial distribution of the FC density in the meta-atom during the photoionization process created using an average irradiance of $\mathrm{I_{avg}^{PEC}} = 2.075\ \mathrm{TW/cm^2}$; the dashed vertical lines enclose the effective hot spot region. **c**. FC density in a horizontal cut plane of the 2D plot in **a** at z=-300 nm.

main text).

$$\begin{aligned}
\mathbf{J}_\mathrm{e}(\mathrm{t}) &= \frac{\partial \mathbf{P}_\mathrm{e}}{\partial \mathrm{t}} = \mathbf{J}_\mathrm{e}^{(1)}(\mathrm{t}) + \mathbf{J}_\mathrm{e}^{(2)}(\mathrm{t}) = -\mathrm{e}\mathrm{N}_\mathrm{e}^{(1)}\mathbf{v}_\mathrm{e}^{(1)}(\mathrm{t}) - \mathrm{e}\int_{-\infty}^{\mathrm{t}} \frac{\partial \mathrm{N}_\mathrm{e}^{(2)}}{\partial \mathrm{t}'}\mathbf{v}_\mathrm{e}^{(2)}(\mathrm{t},\mathrm{t}')\mathrm{d}\mathrm{t}' \\
\mathbf{J}_\mathrm{e}^{(1)}(\mathrm{t}) &= -\frac{\mathrm{e}^2}{\mathrm{m_e}}\mathrm{N}_\mathrm{e}^{(1)}\mathbf{A}(\mathrm{t}); \quad \mathbf{J}_\mathrm{e}^{(2)}(\mathrm{t}) = -\frac{\mathrm{e}^2}{\mathrm{m_e}}\mathrm{N}_\mathrm{e}^{(2)}\mathbf{A}(\mathrm{t}) + \frac{\mathrm{e}^2}{\mathrm{m_e}}\int_{-\infty}^{\mathrm{t}} \frac{\partial \mathrm{N}_\mathrm{e}^{(2)}}{\partial \mathrm{t}'}\mathbf{A}(\mathrm{t}')\mathrm{d}\mathrm{t}'
\end{aligned} \tag{19}$$

Now, we use Poynting's theorem in conjunction with Maxwell's equations to write the time derivative of the total energy density $'\mathrm{U}'$ in a dispersive medium as [11]

$$\begin{aligned}
\frac{\partial \mathrm{U}}{\partial \mathrm{t}} &= \frac{\partial \mathrm{U_{Field}}}{\partial \mathrm{t}} + \frac{\partial \mathrm{U_{Carrier}}}{\partial \mathrm{t}} = -\nabla\cdot(\mathbf{E}\times\mathbf{H}) = \mathbf{E}\cdot(\nabla\times\mathbf{H}) - \mathbf{H}\cdot(\nabla\times\mathbf{E}) \\
&\Rightarrow \frac{\partial \mathrm{U}}{\partial \mathrm{t}} = \frac{\partial}{\partial \mathrm{t}}\left(\frac{1}{2}\epsilon_0\varepsilon_\infty|\mathbf{E}|^2 + \frac{1}{2}\mu_0|\mathbf{H}|^2\right) + \mathbf{E}\cdot\frac{\partial \mathbf{P}_\mathrm{e}}{\partial \mathrm{t}} \\
\mathrm{U_{Field}} &= \frac{1}{2}\epsilon_0\varepsilon_\infty|\mathbf{E}|^2 + \frac{1}{2}\mu_0|\mathbf{H}|^2; \quad \mathrm{U_{Carrier}} = \int_{-\infty}^{\mathrm{t}} \mathbf{E}\cdot\frac{\partial \mathbf{P}_\mathrm{e}}{\partial \mathrm{t}'}\mathrm{d}\mathrm{t}'
\end{aligned} \tag{20}$$

Using $\mathbf{E} = -\partial\mathbf{A}/\partial\mathrm{t}$, and from eq. (19), the expression for $\partial\mathbf{P}_\mathrm{e}/\partial\mathrm{t}$ in the last term on the right side of eq. (20), we get

$$\mathbf{E}\cdot\frac{\partial \mathbf{P}_\mathrm{e}}{\partial \mathrm{t}} = -\frac{\partial \mathbf{A}}{\partial \mathrm{t}}\cdot\mathbf{J}_\mathrm{e} = \frac{\mathrm{e}^2\mathrm{N}_\mathrm{e}^{(1)}}{2\mathrm{m_e}}\frac{\partial|\mathbf{A}|^2}{\partial \mathrm{t}} + \frac{\mathrm{e}^2\mathrm{N}_\mathrm{e}^{(2)}(\mathrm{t})}{2\mathrm{m_e}}\frac{\partial|\mathbf{A}|^2}{\partial \mathrm{t}} - \frac{\mathrm{e}^2}{\mathrm{m_e}}\frac{\partial \mathbf{A}}{\partial \mathrm{t}}\cdot\int_{-\infty}^{\mathrm{t}}\frac{\partial \mathrm{N}_\mathrm{e}^{(2)}}{\partial \mathrm{t}'}\mathbf{A}(\mathrm{t}')\mathrm{d}\mathrm{t}' \tag{21}$$

Integrating the equation above and further simplifying using integration by parts yields

$$\begin{aligned}
\mathrm{U_{Carrier}} = \int_{-\infty}^{\mathrm{t}} \mathbf{E}\cdot\frac{\partial \mathbf{P}_\mathrm{e}}{\partial \mathrm{t}'}\mathrm{d}\mathrm{t}' &= \left[\frac{\mathrm{e}^2\mathrm{N}_\mathrm{e}^{(1)}|\mathbf{A}|^2}{2\mathrm{m_e}} + \frac{\mathrm{e}^2\mathrm{N}_\mathrm{e}^{(2)}(\mathrm{t})|\mathbf{A}|^2}{2\mathrm{m_e}} - \frac{\mathrm{e}^2}{2\mathrm{m_e}}\int_{-\infty}^{\mathrm{t}}\frac{\partial \mathrm{N}_\mathrm{e}^{(2)}}{\partial \mathrm{t}'}|\mathbf{A}(\mathrm{t}')|^2\mathrm{d}\mathrm{t}'\right] \\
&- \left[\mathbf{A}(\mathrm{t})\cdot\int_{-\infty}^{\mathrm{t}}\frac{\partial \mathrm{N}_\mathrm{e}^{(2)}}{\partial \mathrm{t}'}\mathbf{A}(\mathrm{t}')\mathrm{d}\mathrm{t}' - \frac{\mathrm{e}^2}{\mathrm{m_e}}\int_{-\infty}^{\mathrm{t}}\frac{\partial \mathrm{N}_\mathrm{e}^{(2)}}{\partial \mathrm{t}'}|\mathbf{A}(\mathrm{t}')|^2\mathrm{d}\mathrm{t}'\right]
\end{aligned} \tag{22}$$

Further simplifying the above expression, $U_{Carrier}$ can be rewritten as

$$U_{Carrier} = \frac{1}{2}\epsilon_0\omega_p^2|\mathbf{A}|^2 + \int_{-\infty}^{t}\frac{\partial N_e^{(2)}}{\partial t'}\frac{|e\mathbf{A}(t')|^2}{2m_e}dt' - \mathbf{A}(t)\cdot\left(\frac{e^2}{m_e}\int_{-\infty}^{t}\frac{\partial N_e^{(2)}}{\partial t'}\mathbf{A}(t')dt'\right) \tag{23}$$

Integrating eq.(20) and using the expressions for $U_{Carrier}$ from eq. (23), yields

$$\begin{aligned} U = &\frac{1}{2}\epsilon_0|\mathbf{E}|^2 + \frac{1}{2}\mu_0|\mathbf{H}|^2 + \frac{1}{2}\epsilon_0(\varepsilon_\infty - 1)|\mathbf{E}|^2 + \frac{1}{2}\epsilon_0\omega_p^2|\mathbf{A}|^2 \\ &+ \int_{-\infty}^{t}\frac{\partial N_e^{(2)}}{\partial t'}\frac{|e\mathbf{A}(t')|^2}{2m_e}dt' - \mathbf{A}(t)\cdot\left(\frac{e^2}{m_e}\int_{-\infty}^{t}\frac{\partial N_e^{(2)}}{\partial t'}\mathbf{A}(t')dt'\right) \end{aligned} \tag{24}$$

We use eq. (24) to investigate the effects of a TI on total energy in a Drude-Lorentz medium varying in time, where the free electrons that create a TI are generated at rest. A detailed analysis of all the terms in eq. (24) is given in Sections 3.3 and 4.2 of the main text.

S7 Quasistatic Magnetic Field Amplitude

We use Ampere's law (eq. (5) in the main text) along with the expression for the current density from eq. (19) to write

$$\nabla(\nabla\cdot\mathbf{A}) - \nabla^2\mathbf{A} + \frac{\varepsilon_\infty}{c^2}\frac{\partial^2\mathbf{A}}{\partial t^2} + \frac{\omega_p^2}{c^2}\mathbf{A} - \frac{e^2}{\epsilon_0 m_e c^2}\int_{-\infty}^{t}\frac{\partial N_e^{(2)}}{\partial t'}\mathbf{A}(t')dt' = 0 \tag{25}$$

Now, using Coulomb's Gauge ($\nabla\cdot\mathbf{A} = 0$), and the ansatz, $\mathbf{A}(\mathbf{r},t) = \mathbf{A}_s(\mathbf{r}) + \mathbf{A}_t(\mathbf{r},t) + c.c.$, where $\mathbf{A}_t(\mathbf{r},t) = \mathbf{A}_{t0}(\mathbf{r})e^{-i\omega_f t}$, for the magnetic vector potential at a time after the time interface, yields

$$(\omega_p^2 - \varepsilon_\infty\omega_f^2 - c^2\nabla^2)\mathbf{A}_t + (\omega_p^2 - c^2\nabla^2)\mathbf{A}_s = \frac{e^2}{\epsilon_0 m_e}\int_{-\infty}^{t}\frac{\partial N_e^{(2)}}{\partial t'}\mathbf{A}(t')dt' \tag{26}$$

Comparing the time-independent components on the right side of eq. (26) with the left side, we can derive a general differential equation to determine the magnitude of the static magnetic vector potential ($\mathbf{A}_s$) and consequently the static magnetic field ($\mathbf{H}_s$) at the hot spot corresponding to the QS mode as

$$\begin{aligned} (\omega_p^2 - c^2\nabla^2)\mathbf{A}_s(\mathbf{r}) &= \frac{e^2}{\epsilon_0 m_e}\int_{-\infty}^{t}\frac{\partial N_e^{(2)}}{\partial t'}\mathbf{A}(\mathbf{r},t')dt' \\ \mathbf{H}_s(\mathbf{r}) &= \frac{1}{\mu_0}\nabla\times\mathbf{A}_s(\mathbf{r}) \end{aligned} \tag{27}$$

Furthermore, we can write the dispersion relation for propagating electromagnetic waves in the hot spot from eq. (26) as

$$\nabla^2\mathbf{A}_t = \frac{1}{c^2}(\omega_p^2 - \varepsilon_\infty\omega_f^2)\mathbf{A}_t \tag{28}$$

For a homogeneous plasma medium, the dispersion relation in eq. (28) for a monochromatic wave propagating with a wave vector $\mathbf{k}_0$ reduces to the usual dispersion relation of an EM wave in a plasma and gives,

$$\varepsilon_\infty\omega_f^2 = \omega_p^2 + c^2\mathbf{k}_0^2 \tag{29}$$

S8 Theoretical Comparison and Experimental Realization

The Inverse Faraday Effect (IFE) is a well-known magneto-optical effect [12], enabling dynamic-to-static field conversion via nonlinear interactions between a circularly polarized electromagnetic field and the electrons in the nonlinear medium. In this section, we compare several interesting properties of IFE with those of our proposed localized free-carrier generation (LFCG) mechanism.

**Property**	**IFE**	**LFCG**
Material Requirements	Nonlinear Optical Medium ($\chi^{(3)} \neq 0$)	Dielectric medium (No $\chi^{(3)}$ dependence)
EM Field	Circularly Polarized Light	All Polarizations
Rectification Amplitude	Hundreds of mT to a few T [13, 14]	> 1.3 T (higher for a sharper TI and larger field enhancement)

A primary challenge in experimentally demonstrating the proposed mechanism of dynamic-to-static field conversion via localized free-carrier generation is identifying a laser source of sufficient intensity for effective carrier generation, as well as an optical element that can withstand the laser beam without damage while precisely focusing it at the centers of all meta-atoms. However, we can circumvent the problem of localized metallization within each meta-atom by metalizing all meta-atoms simultaneously with a single pump pulse, eliminating the need for a diffractive optical element required for localized metallization and thereby rectifying the complete spatial profile of the resonantly enhanced magnetic field within them. We performed numerical time-domain simulations of complete metallization of all meta-atoms and obtained a maximum amplitude of a magnitude similar to that observed in localized metallization simulations. However, in this scenario, we do not obtain a frequency-shifted metasurface-guided wave because the entire meta-atom array is metalized, completely expelling any oscillating metasurface-guided wave field. The supplementary video shows dynamic-to-static field conversion under complete metallization of the meta-atoms.

Numerical Simulations

All frequency-domain simulations were performed using the COMSOL frequency-domain solver (ewfd) locally on our in-house workstation, which runs 112 threads at 2 GHz with 384 GB of DDR4 system memory at 4800 MT/s. Time-domain simulations of rapid free-carrier generation were performed using the COMSOL time-domain solver (ewt) on the ZEUS workstation of the Laboratory of Plasma Studies at Cornell University. ZEUS runs 128 threads at 2.7 GHz (turbo up to 4 GHz) and has 512 GB of DDR5 RAM running at 4800 MT/s [15]. A complete time-interface simulation with a time interface at 3000 fs and a total simulation time of 5000 fs requires 38 h 55 min 18 s to run on ZEUS using 64 threads.

SOM References

[1] J. D. Joannopoulos, S. G. Johnson, J. N. Winn, and R. D. Meade, *Photonic Crystals: Molding the Flow of Light*. Princeton: Princeton University Press, 1995.

[2] J. C. Slater, "Microwave electronics," *Reviews of Modern Physics*, vol. 18, pp. 441–512, Oct 1946.

[3] T. Ma and G. Shvets, "Scattering-free edge states between heterogeneous photonic topological insulators," *Physical Review B*, vol. 95, p. 165102, Apr 2017.

[4] G. Shvets and Y. A. Urzhumov, "Engineering the electromagnetic properties of periodic nanostructures using electrostatic resonances," *Physical Review Letters*, vol. 93, p. 243902, Dec 2004.

[5] Y. A. Urzhumov and G. Shvets, "Optical magnetism and negative refraction in plasmonic metamaterials," *Solid State Communications*, vol. 146, no. 5, pp. 208–220, 2008.

[6] L. V. Keldysh, "Ionization in the Field of a Strong Electromagnetic Wave," *Journal of Experimental and Theoertical Physics*, vol. 20, no. 5, pp. 1307–1314, 1965.

[7] J. Van Zeghbroeck, *Principles of Semiconductor Devices*. Boulder: Bart Van Zeghbroeck, 2011.

[8] T. Amotchkina, M. Trubetskov, D. Hahner, and V. Pervak, "Characterization of e-beam evaporated Ge, YbF3, ZnS, and LaF3 thin films for laser-oriented coatings," *Applied Optics*, vol. 59, pp. A40–A47, Feb 2020.

[9] T.-T. Yeh, H. Shirai, C.-M. Tu, T. Fuji, T. Kobayashi, and C. Luo, "Ultrafast carrier dynamics in ge by ultra-broadband mid-infrared probe spectroscopy," *Scientific Reports*, vol. 7, p. 40492, 01 2017.

[10] V. E. Gruzdev, "Fundamental mechanisms of laser damage of dielectric crystals by ultrashort pulse: ionization dynamics for the Keldysh model," *Optical Engineering*, vol. 53, no. 12, p. 122515, 2014.

[11] L. Landau and E. Lifshitz, *Electrodynamics of Continuous Media*. Course of theoretical physics, Oxford: Pergamon, 2003.

[12] J. P. van der Ziel, P. S. Pershan, and L. D. Malmstrom, "Optically-induced magnetization resulting from the inverse faraday effect," *Phys. Rev. Lett.*, vol. 15, pp. 190–193, Aug 1965.

[13] O. H.-C. Cheng, D. H. Son, and M. Sheldon, "Light-induced magnetism in plasmonic gold nanoparticles," *Nature Photonics*, vol. 14, pp. 365–368, June 2020.

[14] X. Yang, Y. Mou, B. Gallas, A. Maitre, L. Coolen, and M. Mivelle, "Tesla-range femtosecond pulses of stationary magnetic field, optically generated at the nanoscale in a plasmonic antenna," *ACS Nano*, vol. 16, no. 1, pp. 386–393, 2022. PMID: 34962766.

[15] C. U. Center for Advanced Computing, "Zeus system configuration," 2026. Accessed: June 3, 2026.
